# Physico-chemical properties of waste derived biochar from community scale faecal sludge treatment plants

**DOI:** 10.12688/gatesopenres.13727.2

**Published:** 2022-12-13

**Authors:** Hannah Larissa Nicholas, Ian Mabbett, Henry Apsey, Iain Robertson

**Affiliations:** 1 Department of Chemistry, Faculty of Science and Engineering, Swansea University, Swansea, Wales, SA2 8PP, UK; 2 Department of Geography, Faculty of Science and Engineering, Swansea University, Swansea, Wales, SA2 8PP, UK

**Keywords:** Biochar, Faecal Sludge, Fecal, Characterization, Properties, Pyrolysis, Sanitation

## Abstract

**Background**: The dumping of untreated faecal sludge from non-sewered onsite sanitation facilities causes environmental pollution and exacerbates poor public health outcomes across developing nations. Long-term mechanisms to treat faecal sludge generated from these facilities are needed to resolve the global sanitation crisis and realize the Sustainable Development Goal (SDG) 6 “ensure availability and sustainable management of water and sanitation for all” by 2030.  Pyrolysis of faecal sludge removes pathogens and generates biochar, which can be used as a soil enhancer.

**Methods:** The properties of faecal sludge biochars from three full-scale treatment plants in India were determined via Fourier transform infrared (FTIR) spectroscopy, scanning electron microscopy (SEM), energy dispersive x-ray (EDX) spectroscopy, crystal x-ray diffraction (XRD), proximate analyses, and BET surface area porosimetry.

**Results: ** Results showed that all three biochars had low specific surface area, high alkaline pH values, high ash content, and negative surface charge. Fourier transform infrared spectra showed the same surface functional groups present in each biochar. X-ray diffraction analysis showed the mineral composition of each biochar differed slightly. Scanning electron microscopy analysis indicated a porous structure of each biochar with ash particles evident.

**Conclusions: **Slight differences in the ash content, surface area, pH and mineral content was observed between the three biochars.

## Article highlights

- The physico-chemical properties of faecal sludge biochars from full-scale pyrolysis facilities were evaluated
- Biochars recorded very alkaline pH values, high ash content, low carbon content, low specific surface area
- Similar FTIR spectra indicated the same functional organic groups present on the biochars surface
- There were differences in ash content, pH and mineral content between the biochars

## Introduction

Improving sanitation along with hygiene practices and access to safe water are essential for improving socioeconomic development and health globally. Inadequate sanitation facilities and lack of clean water are key factors in the contraction of diarrhoeal diseases world-wide leading to 1.6–2.5 million deaths every year ( Kosek *et al.*, 2003).

In 2000 the international community set out eight Millennium Development Goals, a template aimed at tackling the needs of the world’s most poverty stricken and underprivileged ( Hutton & Bartram, 2008). Target 7C of the Environmental Sustainability Goal was to reduce by half the proportion of citizens “without sustainable access to safe drinking water and basic sanitation”. Since 2000, however, the proportion of the population in low and middle- income nations that use “unimproved” sanitation facilities has increased ( WHO & UNICEF, 2017).

In 2015 the international community set out 17 new Sustainable Development Goals including Goal 6, to “ensure availability and sustainable management of water and sanitation for all” ( UN, 2015). It is estimated that 3.6 billion people in the world still do not have access to safely managed sanitation facilities ( UNICEF/WHO, 2021). Approximately 2.1 – 2.6 billion of these depend on onsite sanitation facilities ( UNICEF & WHO, 2017) that generate vast quantities of untreated faecal sludge each day. In developing countries, faecal sludge (FS) collected from onsite sanitation facilities has been poorly managed, which has led to negative public and environmental health outcomes from eutrophication of surface water bodies, and contamination of groundwater and soils ( Gwenzi & Munondo, 2008), and poor social and economic development ( Haller *et al.*, 2007; Mara *et al.*, 2010). Long term and more sustainable solutions to deal with faecal sludge that don’t involve expensive, water intensive and energy intensive sewer systems are needed.

Recent research has focused upon thermochemical treatment, with an emphasis on pyrolysis as a safe method of disposing of faecal sludge ( Krueger *et al.*, 2020). Pyrolysis is the heating of biomass to temperatures of 350°C – 1000°C in an oxygen-free environment ( European Biochar Foundation, 2016) which eliminates harmful pathogenic organisms within the sludge. Carbon-rich biochar produced from pyrolysis does not readily burn like charcoal ( Crombie *et al.*, 2013), is safe to handle, and has been demonstrated to be an important soil amendment ( Chan *et al.*, 2007). The original feedstock source, pyrolysis temperature, hold time, and heating rate are the main factors determining the characteristics of biochars ( Chen *et al.*, 2008; Crombie *et al.*, 2015; Lehmann & Joseph, 2012; Tomczyk *et al.*, 2020; Weber & Quicker, 2018).

The theory behind the utilization of biochar to improve soil fertility and increase crop yield originated from observations made on the Amazonian Black Earth ( *Terra Preta*). *Terra Preta* is a specific type of very dark, fertile soil discovered in the Amazon basin, containing higher nutrient levels and higher organic carbon content than the surrounding soils which are generally low in fertility ( Glaser *et al.*, 2001).

There are multiple benefits to adding biochar to soil aside from improving carbon content and nutrient levels. Surface functional groups on the surface of biochar can lead to an increase in the cation exchange capacity of the soil (CEC) ( Glaser *et al.*, 2001); the microporous structure of biochar can increase the water holding capacity of the soil ( Gaskin *et al.*, 2007), and alkaline biochars can increase pH levels in acidic soil ( Novak *et al.*, 2009).

Biochar addition to soil has also been shown to reduce the bioavailability of heavy metals in soils ( Park *et al.*, 2011; Uchimiya *et al.*, 2011; Zhang *et al.*, 2013) including that of wastewater sludge biochar ( Hossain *et al.*, 2010) and sewage sludge biochar ( Méndez *et al.*, 2012). Biochar addition also reduces plant uptake of pesticides ( Yu *et al.*, 2009) and reduces the leaching of applied pesticides which can impact underground water contamination ( Ahmad *et al.*, 2014). Changes in soil microbial properties upon biochar addition have also been reported ( Lehmann *et al.*, 2011), including alteration of soil microbial community structure ( Farrell *et al.*, 2013), and an increase in microbial abundance ( Awad *et al.*, 2018).

There is a considerable amount of research investigating characteristics of sewage sludge-derived biochar but less on faecal sludge biochar ( Gold *et al.*, 2018). Many studies exist on the properties of faecal sludge itself ( Awere *et al.*, 2020; Bassan *et al.*, 2013; Fanyin-Martin *et al.*, 2017; Lama *et al.*, 2022; Schoebitz *et al.*, 2014; Septien *et al.*, 2020). Only a handful of articles examine properties of faecal sludge biochar and these studies have a diverse range of objectives including biochar as a soil amendment to increase lettuce yields ( Woldetsadik *et al.*, 2018), biochars solid fuel characteristics ( Krueger *et al.*, 2020), cadmium adsorption by biochar ( Koetlisi & Muchaonyerwa, 2017), recovery of ammonium in urine by biochar ( Bai *et al.*, 2018) and energy balance analysis of slow pyrolysis of human manure ( Liu *et al.*, 2014).

Most of the research into faecal sludge-derived biochar has focused on characterization of small-scale laboratory-produced biochar ( Bleuler *et al.*, 2021; Gold *et al.*, 2018; Liu *et al.*, 2014; Woldetsadik *et al.*, 2018) while data from full-scale operations are very limited ( Krueger *et al.*, 2020). Investigating the feasibility of resource recovery of operational up-scaled sludge treatment technologies and production of FS biochar with consistent properties is imperative to alleviate the sanitation crisis ( Andriessen *et al.*, 2019; Strande *et al.*, 2014). Krueger *et al.* (2020) investigated the physico-chemical properties of full-scale faecal sludge biochars from treatment plants in Warangal and Narsapur, India. They focused on solid fuel properties of biochar, particle size distribution and heavy metal concentration. Heavy metal concentrations were found to be within the limits for land application set out by the EU ( EEC, 1986) and the International Biochar Initiative ( IBI, 2015) apart from the Narsapur biochar which contained concentrations of lead over the IBI stated threshold.

The objective of this investigation was to assess the uniformity of biochar characteristics produced from three full-scale faecal sludge treatment plants in Wai, Warangal and Narsapur, India. This study focused more on physico-chemical properties that would contribute to biochars end-use as a soil amendment. The biochar properties determined were ash content, pH, carbon content, organic surface groups, surface charge, mineral content, pore volume, and specific surface area.

## Methods

### Biochar preparation

**Figure 1.**
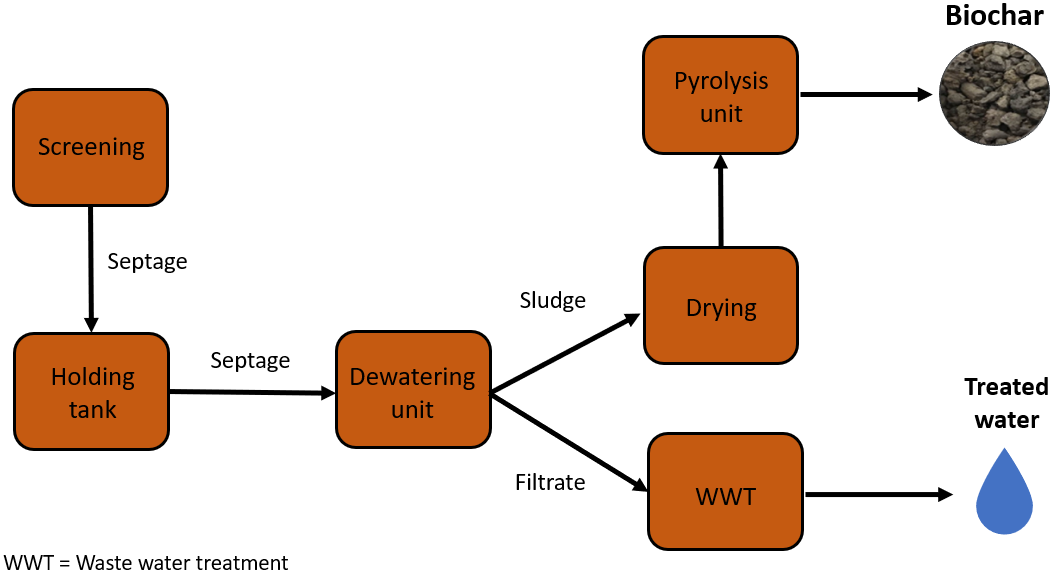
Flow diagram of waste through a Tide Technocrats Community Scale Faecal Sludge and Septage Processor adapted from ( Tide Technocrats, n.d).

**Figure 2.**
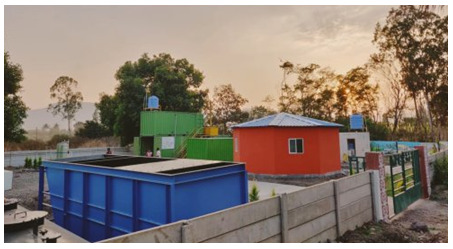
Photo of the community scale faecal sludge and septage treatment plant in Wai, Maharashtra ( Tide Technocrats, n.d.).

The faecal feedstocks for the preparation of the biochars used in this study were sourced from three different faecal sludge and septage processors in India: Narsapur in Andhra Pradesh, Warangal in Telangana and Wai, Maharashtra ( Figure 2). Warangal and Narsapur treatment plants currently have a capacity of 15m ^3^ per day, whereas the Wai treatment plant has a capacity of 70 m ^3^ per day. FS collected from septic tanks is delivered to each processing plant where it is stored in holding tanks for the homogenization of the sludge. Tide Technocrats Private Limited have several community scale faecal sludges and septage processors which sanitize faecal waste and dewaters the sludge (5–10% moisture content) using solar energy in drying beds. Solar drying was managed on-site and expedited by spreading the sludge in a 10 mm layer. The sludge was pyrolyzed into biochar using a flame temperature operating range of 550–750°C. The process relies on autothermal operation, thus a limited supply of oxygen flows through an air fan into the main reaction chamber to allow for partial oxidation. process at the community scale faecal sludge and septage processors are outlined in Figure 1. Three 5kg biochar samples were collected from each processor in September 2018.

### Characterization of biochars

The biochars characterized were collected from the pyrolyzer.

***Chemical analysis.*** Elemental C, N, S and H abundances were determined at Environmental Geosciences, University of Vienna, Austria using an elemental analyzer (Vario MACRO, Elementar). 

The elemental composition (C, H, N, S and O) and ash content of the biochars were used to calculate the molar element ratios H/C, C/N, and O/C. The amount of oxygen in the samples was calculated from the subtraction of total percentage carbon, hydrogen, nitrogen, and sulphur and ash content from 100 ( Castan *et al.*, 2019).

***Proximate analyses.*** Moisture and ash content of the three biochars were determined in triplicate by methods adapted from the literature ( ASTM D 1762-84, 2011; Enders *et al.*, 2012).

Crucibles and covers were cleaned by heating at 750°C for 6 hours and then cooling to 105°C. This procedure volatilized residual material on the crucibles. The crucibles were transferred to desiccators and cooled to ambient temperature. The mass of crucibles and crucible covers were recorded to 0.1mg and masses determined for all samples. Approximately 1.0g of biochar was added into each crucible. For the moisture determination the crucibles and covers were heated at 105°C for 18 hours and then transferred to desiccators whilst hot. The covers were removed briefly in order to safely remove the crucibles and covers from the oven. After cooling to ambient temperature, the mass of crucibles, covers and sample were recorded to 0.1mg for all samples.

For ash determination the covered crucibles with 105°C dry biochar was placed in the furnace. The covers were adjusted so that they were askew to allow air flow into the crucibles, while reducing the possibility of physical losses. The samples were heated from ambient to 750°C at a rate of 2°C per minute. The furnace was programmed to hold the temperature at 750°C for 6 hours then allowed to cool down to ~130°C. Crucible lids were adjusted to sit flush when the temperature of 105°C was reached. The crucibles were then removed from the furnace before placing in desiccators and left to cool to ambient temperature. The mass of each crucible and crucible cover with sample was recorded to 0.1mg.

Chars were ground to <850µm in a pestle and mortar to enhance representativeness of the sample and sieved to >149µm as this lessens physical losses upon rapid heating ( Enders & Lehmann, 2015).

***pH and electrical conductivity.*** The pH of biochar samples was measured by suspending 5.0g (ground to <2mm) biochar in deionised water in a 1:10 ratio ( Singh *et al.*, 2017). After 1 hour of shaking, suspensions were allowed to stand for 30 minutes before pH measurements were taken using a Voltcraft soil pH meter calibrated using pH 7 and pH 10 buffers. Electrical conductivity (EC) was measured on the same samples using a calibrated Whatman CDM 400 EC meter. The analyses of pH and EC were performed in triplicate.

***FT-IR analysis.*** Fourier transform infrared (FTIR) spectra were used to identify the surface organic functional groups present in the biochar. High ash content in sludge-derived biochars leads to a high mineral content with bands in the infrared spectrum arising at similar wavenumbers to organic functional groups. To elucidate the different groups present, FTIR spectra of ashed biochars and de-ashed (acid washed) biochars were also generated.

Acid washing biochar to remove ash content ( Klasson *et al.*, 2009; Lima *et al.*, 2016; Thomas Klasson *et al.*, 2014) was achieved with 0.1 M HCl at a ratio of approximately 50:1 (v/w). Samples were shaken in a Uniwist 400 at 180 rpm for 2 hours before being filtered and washed with deionised water until a pH of 7 was reached. Samples were oven-dried at 80°C overnight.

The samples were gently ground using a pestle and mortar and analyzed using a Perkin Elmer Spectrum 2 FTIR spectrophotometer applying the Attenuated Total Reflectance (ATR) method with a diamond crystal. The resulting spectra were an average of 16 scans obtained in the range from 400 to 4000 cm ^−1^ with a spectral resolution of 2 cm ^−1^ for biochars and 4 cm ^−1^ for acid washed and ashed biochars.

***Surface area.*** The BET (Brunauer, Emmett, and Teller) method is frequently used to determine the total surface area and pore size of materials. The BET analysis was conducted using the NOVA 2200e surface area and pore size analyzer (Quantachrome Instruments). The BET specific surface area of the three biochar samples were determined using two methods: N _2_ as adsorptive gas at 77 K and CO _2_ at 273 K.

Prior to these measurements, 200mg – 300mg of biochar (<2mm) were heated to 130°C under vacuum for a minimum of 4 hours. Then the samples were transferred to the instrument and outgassed at 105°C for a minimum of 4 hours following standard protocols. Samples were analyzed in triplicates.

For N _2_ isotherms and CO _2_ isotherms the BET equation was used to determine the specific surface areas from six points in the pressure region P/P _0_ = 0.01–0.30 ( Brunauer *et al.*, 1938). For N _2_ the pore size-distributions in the pressure region P/P _0_ = 0.01–0.98 were ascertained using the built-in Density Functional Theory (DFT) model assuming slit-like pores. DFT considers micropore filling process, the development of the adsorbed film thickness, and importantly capillary condensation and evaporation, thus it can model hysteresis in the adsorption/desorption mesopore region of the isotherm.

For CO _2_ isotherms the pore size-distribution, the cumulative pore volume (μPV) and the cumulative surface area (μSSA) in the pressure region P/P _0_ = 0.001–0.030 were determined using the built-in Grand Canonical Monte Carlo (GCMC) simulation, again, assuming pores were slit-shaped.

The benefit of using of DFT and Monte Carlo simulation methods is that they provide a combined micro-mesopore analysis.

***Measurement of zeta potential.*** Zeta potential measurements were undertaken according to methods reported in the literature ( Samsuri *et al.*, 2013; Yuan *et al.*, 2011). The zeta potential values were determined by weighing 0.045g of 63µm sieved biochar into a 250ml conical flask and adding 180 mL of 0.1M NaCl solution to each flask. Five suspensions were prepared for each biochar at pH values between 5.0 – 9.0 with the pH of each suspension adjusted using HCl . Suspensions were then dispersed ultrasonically for 30 minutes at 30 ± 1 °C in a bath-type sonicator at a frequency of 40 kHz and a power of 300 W. The samples were then left to stand for 72 hours before being measured with a Malvern Zeta Sizer Nano. In total 15 suspensions were prepared and each suspension was measured a minimum of three times.

***X-ray diffraction (XRD).*** The X-ray diffraction (XRD) analysis of the chars was conducted on a Bruker D8 Discover XRD. This was operated at 40 kV and 40 mA and the data collected over a 2 *θ* range of 20–70° using the Cu-Kα radiation at a scan rate of 2° min ^−1^. The main phase peaks were identified by comparing the observed XRD patterns to the standards compiled by the Crystallography Open Database (COD) ( Downs & Hall-Wallace, 2003; Gražulis *et al.*, 2009; Gražulis *et al.*, 2015, Gražulis *et al.*, 2012; Merkys *et al.*, 2016; Quirós *et al.*, 2018).

***SEM/EDX.*** SEM–EDX analysis offers detailed imaging data about the morphology and surface texture of individual particles, with characterization of the elemental composition of the analyzed volume. Scanning electron microscope (SEM) analysis was performed using a Hitachi TM3000 SEM fitted with a Bruker X-ray energy dispersive spectrometry (EDS). The two modes of operation in SEM analysis utilized here were backscattered electron imaging (BSE) and energy dispersive x-ray EDX. Biochar particles used were in the size range 150 µm– 850 µm. Prior to analysis samples were spread onto double-sided carbon tape and mounted on a SEM stub.

***Cation exchange capacity.*** Cation exchange capacity measurements were performed in University of Santiago de Compostela, Spain, by the summation method of the exchangeable base cations of Ca, Mg, Na, K, and Al. NH _4_Cl 1M (25ml) was added to the biochar sample (5g) and shaken manually before being left to stand overnight (16 hours). The following day, 75 ml of NH _4_Cl 1M was added and then filtered using quantitative, low ash filter paper. ( Peech *et al.*, 1947). Ca, Mg and Al were measured by PerkinElmer PinAAcle 500 Atomic Absorption Spectrometer and Na, K, were measured by Atomic Absorption Spectophotometer with an Emission Flame.

### Statistical analysis

Electrical conductivity, pH, moisture content, ash content, CEC, surface area and pore volume measurements were all performed in triplicate and experimental uncertainty given by standard deviation.

## Results

### Proximate analyses EC, pH and elemental analyses

All biochars collected from the pyrolyzer had high ash contents (Nicholas, 2022). Warangal biochar (WGL_BC) recorded the highest ash content at 88.3% and Narsapur biochar (NSP_BC) and Wai biochar (WAI_BC) had lower ash contents at 67.0% and 62.3% respectively ( Table 1.). Warangal biochar (WGL_BC) also had the lowest moisture content at 0.98% in comparison with 2.15% and 3.08% for Narsapur biochar (NSP_BC) and Wai biochar (WAI_BC) respectively. Measured pH values were high for all three biochars (11.86 – 12.45). The measured high ash content is consistent with the literature ( Gold *et al.*, 2018; Koetlisi & Muchaonyerwa, 2017; Liu *et al.*, 2014). The initial feedstock of sewage sludge is high in ash and sewage sludges have been found to contain very high concentrations of Si (19–58%), Ca (5.1–7.4%), and P (3.4–4.9%) ( Zielińska *et al.*, 2015). Ash content of faecal sludge is also high and has been measured at 17.0 wt.%, significantly higher than measured ash content of sawdust at 0.8% ( Liu *et al.*, 2014). Ash content of faecal sludge has been found to be higher than that of sewage sludge ( Koetlisi & Muchaonyerwa, 2017). Several reasons for this high ash content have been suggested including loss of volatile solids from latrine waste due to long storage times in the latrine ( Zuma *et al.*, 2015), ingress of grit and sand from poorly lined containment structures ( Niwagaba *et al.*, 2014) and in community toilets a higher rate of disposal of polluting waste can lead to higher ash contents ( Barani *et al.*, 2018).

**Table 1.** Proximate analyses, elemental analyses, pH, EC and surface area measurements of faecal sludge biochars. (EC = Electrical Conductivity, C= Carbon, N= Nitrogen, S= Sulphur, Oxygen, SBET = Surface area measured by BET, TPV = Total pore volume, SSA = Specific Surface area, CEC=Cation Exchange Capacity).

Parameter	Unit	WAI BC	NSP BC	WGL BC
pH	[ ]	11.81 ± 0.01	11.82 ± 0.01	12.45 ± 0.01
EC	[mS.cm ^-1^]	2.70 ± 0.09	1.79 ± 0.17	9.00 ± 0.02
Moisture	[%]	3.08 ± 0.01	2.15 ± 0.31	0.98 ± 0.05
Ash	[%]	62.3 ± 0.32	67.0 ± 2.68	88.3 ± 0.21
C	[%]	21.11	23.79	8.06
N	[%]	1.32	1.13	0.37
H	[%]	1.55	0.73	1.15
S	[%]	0.03	0.27	0.03
O	[%]	13.69	7.08	2.09
H/C	[ ]	0.9	0.4	1.7
C/N	[ ]	18.7	24.6	25.4
O/C	[ ]	0.5	0.2	0.2
S _BET_ N _2_	[m ^2^.g ^-1^]	3.52 ± 0.78	3.69 ± 0.36	12.07 ± 4.12
N _2_ TPV	[cm ^3^.g ^-1^]	0.011	0.011	0.019
S _BET_ CO _2_	[m ^2^.g ^-1^]	46.72 ± 7.0	74.20 ± 4.0	26.11 ± 2.6
CO _2_ µSSA	[m ^2^.g-1]	63.49 ± 8.3	99.62 ± 4.5	36.76 ± 3.0
CO _2_ µPV	[cm ^3^.g ^-1^]	0.017	0.027	0.010
CEC	[cmol.kg ^-1^]	90.0 ± 6.5	41.9 ± 2.2	129.3 ± 2.3

Increases in pH due to increases in ash content in biochars derived from sewage sludge feedstocks have been previously reported ( Hossain *et al.*, 2011; Liu *et al.*, 2014). The general alkaline character of biochar likely results from the increase in quantities of alkali salts (Na, K) and salts of alkaline elements (Ca, Mg) during the pyrolysis process ( Singh *et al.*, 2010).

WGL_BC recorded the most alkaline pH value (12.45) and the largest EC value ( Table 1.) which is due to the higher ash content recorded for WGL_BC ( Rehrah *et al.*, 2014). There could be several reasons why the WGL biochar had a significantly higher ash content. Digestion during storage in onsite sanitation technologies can play a part in the high ash content of FS biochar ( Gold *et al.*, 2018) as well as contamination of FS by sand and grit caused by poorly lined containment structures ( Niwagaba *et al.*, 2014). A recent study investigating biochar from the same treatment facilities in India observed that sintered mineral depositions had to be removed from the reactor on a weekly basis ( Krueger *et al.*, 2020). Therefore, it is likely that ash concentrations from biochars from these types of treatment plants will fluctuate over time.

A high ash content of biochar could be useful with regards to its end-use a soil amendment. Increased crop growth with a highly alkaline (12.1), high ash biochar treatment of acidic soil has been previously reported ( Smider & Singh, 2014) The authors deemed this was a result of the release of nutrients from the ash in the biochar itself and the biochar’s liming effect. It has been proposed that this liming effect is one of the main processes influencing the enhanced plant growth seen on biochar addition to soils ( Jeffery *et al.*, 2011). Altering soil pH is one of several mechanisms by which biochar can improve soils and increase agricultural productivity. Therefore, highly alkaline biochars could be of benefit to acidic soils are responsible for the severe limitation of crop agriculture worldwide. Currently only a small fraction of acidic soil is used for arable crops globally but approximately 50% of the earth’s potential arable lands are acidic ( von Uexküll & Mutert, 1995).

The elemental composition *( Table 1.)* shows a relatively low percentage of carbon within the samples, 21–23% for NSP_BC and WAI_BC and a very low 8% for WGL_BC which is consistent with the measured ash content. Pyrolysis generally concentrates carbon in the biochar with an increase in C content relative to the feedstock frequently reported. However, most studies on sewage sludge (SS) –derived biochar show a decrease in the percentage of C in the final product relative to the feedstock ( Agrafioti *et al.*, 2013; Khan *et al.*, 2013). FS- and SS–derived biochars generally have low total C concentrations in comparison with cellulose derived biochars ( Tomczyk *et al.*, 2020). This is due to the high ash content in the original feedstock of faecal and sewage sludge. The measured carbon concentrations in these biochars are consistent with carbon contents reported in the literature for faecal sludge biochar 27.4– 34.9% ( Gold *et al.*, 2018), 17.2 – 34.1% ( Krueger *et al.*, 2020), 6.5 – 11.1% ( Koetlisi & Muchaonyerwa, 2017), and 19.5% ( Woldetsadik *et al.*, 2018).

### Fourier transform infrared (FTIR) spectroscopy

**Figure 3.**
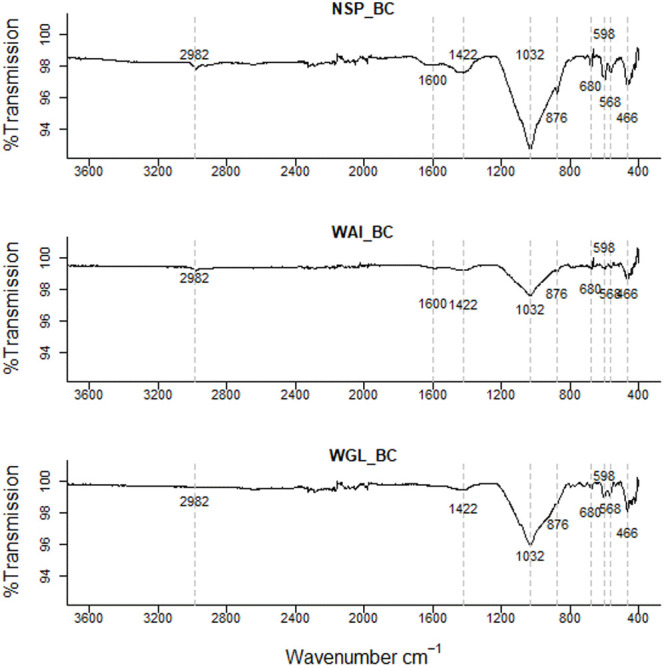
Fourier-transform infrared spectra (FTIR) of the three biochars NSP, WGL and WAI with dotted lines representing the main absorption (cm ^-1^) peaks.

FTIR spectra indicated that all three sludge biochars have a complex chemical bond structure with both organic matter and mineral compounds evident within the biochar. The FTIR spectra of all three biochars were similar with the same functional groups present on the surface; an indication of the homogenous nature of faecal sludge ( Figure 3).

High ash content in sludge-derived biochars leads to a high mineral content with bands arising on the spectrum at similar wavenumbers to organic functional groups. For example, a broad peak in the 1000–1200cm ^-1^ region can arise due to several functional groups such as inorganic and organic silicon, phosphorus compounds, as well as C-O stretching and sulphate groups ( Coates, 2004)

Low intensity peaks evident in the 3800cm ^−1^ –3600cm ^−1^ region relate to OH group vibrations within mineral matter ( Hossain *et al.*, 2011) which indicates the presence of clay type compounds within the biochar ( Table 2). Two peaks at 2980cm ^-1^ and 2890cm ^-1^ indicate asymmetric and symmetric aliphatic ν(CH) from terminal –CH _3_ groups respectively ( Socrates, 2001). However, these CH bands disappear at high temperatures due to demethylation and dehydration ( Zhang *et al.*, 2015) therefore in biochar pyrolyzed at 550 – 750°C the peaks are negligible. 

**Table 2.** Proposed band assignments of the FTIR spectra of biochar.

Wavenumbers (cm ^-1^)	Characteristic vibrations	Reference
3670 - 3650	ν(OH) from non-hydrogen bonded O-H groups	( Sharma *et al.*, 2004)
3600 - 3200	ν(OH) from sorbed water and hydrogen-bonded biochar O-H groups	( Keiluweit *et al.*, 2010)
~2980	2990-2950 cm ^-1^ asymmetric aliphatic v(CH) from terminal –CH _3_ groups	( Socrates, 2001)
~2890	2870-2890 cm ^-1 symmetric^ aliphatic v(CH) from terminal –CH _3_ groups	( Socrates, 2001)
2700-2100	P-OH groups produce one or two broad bands in the 2700 -2100 region 2100 - 2250cm ^-1^ C≡C bonds 2100 - 2360cm ^1^ Silane Si-H 2100 - 2270cm ^-1^ Dimides, Azides and Ketenes	( Stuart, 2004)
1700	v(C=O) from carboxylic acids amides, esters and ketones 1740- 1650	( Socrates, 2001)
1540 - 1650	C==O stretching vibrations for amides, aromatic C=C stretching and carboxylate anion vibrations.	( Calderón *et al.*, 2006)
1580 - 1600	vibration of C=C bonds	( Davis *et al.*, 1999)
1424	Carbonate (ν _3_; asymmetric stretch)	( Socrates, 2001)
1200- 950	P–O (asymmetric and symmetric stretching of PO _2_ and P(OH) _2_ in phosphate)	( Jiang *et al.*, 2004)
1100-1000	Si-O-Si asymmetric stretching	( Falaras, 1999)
1020 - 1030	C–O stretching of ethers and primary amine C–N stretches	( Keiluweit *et al.*, 2010) ( Claoston *et al.*, 2014)
~875	Out-of-plane bending for CO _3_^2−^ and – v(M-O-H) O-H bending bands from clay minerals associated with biochar	( Zhao *et al.*, 2013) ( Farmer, 1974)
796 and 780	quartz doublet	( Farmer, 1974)
462-464	Si-O-Si	( Qian & Chen, 2013)
452	Si-O rocking	Shahrokh Abadi *et al.*, 2015

Small peaks in the 2700–2100 region could be due to P-OH groups in phosphorus acids and esters which produce one or two broad bands ( Stuart, 2004).

A peak at 1424cm ^−1^ in the biochar spectra ( Figure 3.) corresponds to asymmetric stretches of carbonate groups, which correlates with the small peak at 874 cm ^−1^ due to the out-of-plane bending for CO _3_^2−^ ( Zhao *et al.*, 2013). This could indicate the presence of calcite (calcium carbonate) in the sample. The presence of carbonate was verified as the FTIR spectrum of the acid washed biochar showed no clear peaks at 1424 cm ^−1^ or 874 cm ^−1^ confirming that acid washing removed carbonates from the sample ( Figure 5.).

**Figure 4.**
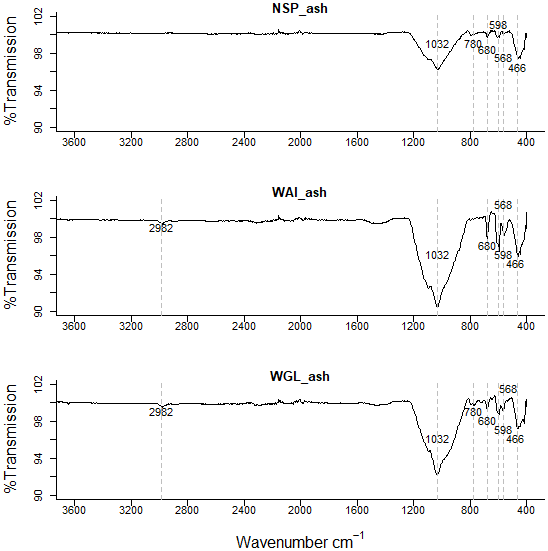
FTIR spectra of ashed NSP biochar, ashed WAI biochar, and ashed WGL biochar.

Another interesting difference between the ashed ( Figure 4.) biochars and deashed biochars is a broad trough between 3400cm ^-1^ and 2500 cm ^-1^. This implies the presence of O-H in carboxylic acids however there is only a very weak intensity peak at ~1700 cm ^-1^ which could correspond to C=O in carboxylic acids. Other possible groups responsible for peaks within the 3400cm ^-1^ and 2500 cm ^-1^ region include ν(OH) from sorbed water and hydrogen bonded OH ( Keiluweit *et al.*, 2010).

**Figure 5.**
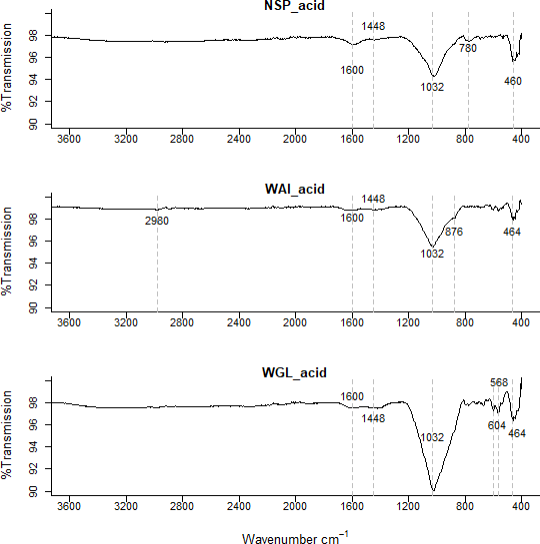
FTIR spectra of deashed (acid washed) NSP, WAI and WGL biochar.

The low intensity peak in biochar between 1540 and 1650 could be indicative of C=O stretching vibrations for amides ( Calderón *et al.*, 2006), aromatic C=C stretching and carboxylate anion vibrations ( Deacon & Phillips, 1980). The peak in the deashed biochar at 1580 cm ^−1^ to 1600 cm ^−1 ^is indicative of a carboxylate ion, the conjugate base of a carboxylic acid ( Deacon & Phillips, 1980; Ellerbrock & Gerke, 2021).

This peak was not evident in the ashed biochar ( Figure 4.). It’s been suggested that a reduction in inorganics by acid demineralization allows previously hidden carbon to emerge so increasing the amount of acidic functional groups ( Lou *et al.*, 2011). In the ashed biochar there are very visible peaks ~1450cm ^-1^ indicative of a carbonate stretch (CO _3_^ 2-^) whereas as the peaks in the acid washed samples are much less visible indicating some carbonate salts within the ash content have been successfully removed by acid demineralization.

The very broad band in the range 1200–970cm ^-1^ is indicative of several functional groups. Inorganic and organic silicon and phosphorus compounds, as well as carbohydrates and sulphates can contribute to this broad peak ( Wen *et al.*, 2007). Sewage chars are known to contain high phosphorus levels suggesting that the peaks observed in 1200–950cm ^-1^ band arise from P containing functional groups such as asymmetric and symmetric stretching of PO _2_ and P(OH) _2_ in phosphate ( Jiang *et al.*, 2004). Si-O asymmetric stretching could also be present between 1000–1100cm ^-1^ ( Falaras, 1999) as well as symmetric C-O stretching of ethers.

A peak at 462–464 cm ^−1^ evident in both biochar and acid washed biochar is indicative of bending vibration of Si-O-Si (459–463 cm ^−1^) ( Qian & Chen, 2013). In the ashed biochar this peak seems to shift to a lower wavenumber 456cm ^-1^. It is possible the signals at 462–464 cm ^−1^ relate to bending vibration of Si-O-Si (459–463 cm ^−1^) and the signal at lower wavelength in the ashed biochar at 452cm ^−1^ relates to Si-O rocking ( Shahrokh Abadi *et al.*, 2015). A weak intensity signal at 1984 cm ^-1^ is evident in the ashed biochar but not in the deashed samples. This signal could indicate metal – carbonyl bonds, typically terminal M-CO bonds occur at 2125 - 1850 cm ^-1^. A quartz doublet at 796cm ^-1^ and 780cm ^-1^ is evident in the ashed biochar sample ( Farmer, 1974).

There are more signals recorded in the 900-400 cm ^-1^ region for the ashed biochar than the deashed biochar which relate to clay minerals associated with biochar. Bands below 600 cm ^–1^ can be caused by stretching inorganic compounds such as KCl and CaCl _2_ ( Hossain *et al.*, 2011).

The oxygen containing functional groups (OCFGs) present on biochars surface such as C=O groups determine its cation exchange capacity (CEC) ( Banik *et al.*, 2018). It is this property that enables biochars to adsorb cationic nutrients such as NH _4_^+^, Ca ^2+^, K ^+^ within the soil and increases soils nutrient retention capability. The lack of C=O groups present in WGL_BC could affect its ability to retain nutrients and therefore its suitability as a soil amendment.

### Surface area

The shape of the isotherms indicate a Type II isotherm, however, Type II isotherms are generally typified by a lack of hysteresis and no saturation at P/P _0_ near to 1; typical of nonporous and macroporous adsorbents ( Thommes *et al.*, 2015). A deviation from a true Type II isotherm can be described as a pseudo-type II isotherm. These isotherms are associated with delayed capillary condensation due to the small degree of pore curvature and non-rigidity of the aggregate structure of the adsorbent. ( Sing & Williams, 2004).

Hysteresis is present in all isotherms and can be classified as either type H3 hysteresis loop or type H4 hysteresis loop according to International Union of Pure and Applied Chemistry ( Thommes *et al.*, 2015). Hysteresis is caused by capillary condensation and is typical of mesoporous materials. H3 and H4 loops do not tend to close until equilibrium pressure is at or close to saturation pressure. H3 type is typical for loose aggregates of plate-like particles and in porous materials typical of pore networks containing macropores not entirely filled with condensate. H4 type loops suggest presence of slit-shaped pores including pores in the micropore region and plate-like particles with spaces between the parallel plates ( Mokaya & Jones, 1995) and are common with activated carbons. H4 hysteresis loops are commonly observed with more complex materials consisting of both micropores and mesopores.

**Figure 6.**
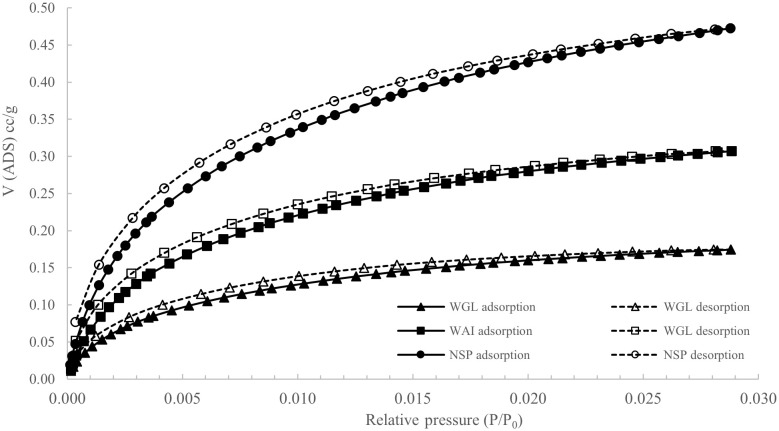
N _2_ adsorption and desorption isotherms of WAI, WGL and NSP biochars (P/P _0_= Relative pressure, V (ADS) cc/g = Volume of adsorption cc/g).

Adsorption and desorption N _2_ isotherms for all biochars ( Figure 6.) showed low surface areas of between 3.52 – 12.07m ^2^g ^-1^ ( Table 1) consistent with results reported in the literature for sewage sludge biochars which have low surface areas due to high ash content ( Agrafioti *et al.*, 2013; Bagreev *et al.*, 2001; Schimmelpfennig & Glaser, 2012). It has been postulated that high ash contents reduce surface area by filling or blocking access to the biochar micropores ( Song & Guo, 2012). 

**Figure 7.**
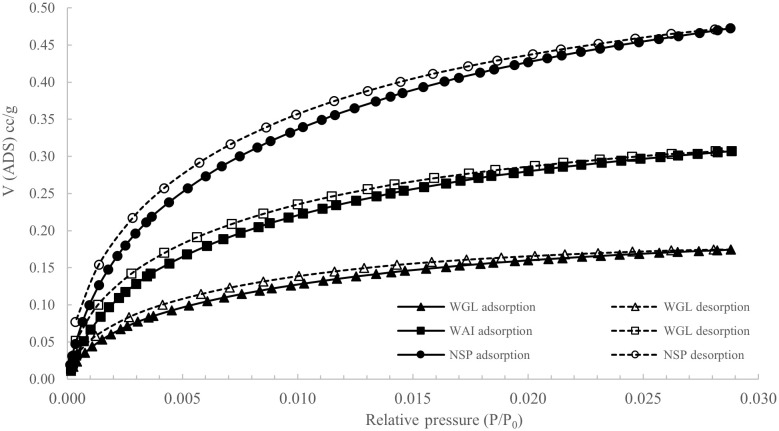
CO _2 _adsorption and desorption isotherms for WAI, WGL and NSP biochars (P/P _0_= Relative pressure, V (ADS) cc/g = Volume of adsorption cc/g).

The low nitrogen uptake of all three biochars can be characteristic of materials with small ultra-micropores that are close to the kinetic diameters of nitrogen, since molecules cannot overcome the activation energy for passing through the pores at cryogenic temperatures ( Kim *et al.*, 2011). To investigate this potential microporosity further CO _2_ adsorption isotherms at 273K were recorded for the three biochar samples ( Figure 7).

The CO _2_-based BET specific surface areas (S _BET_, μSSA) and pore volume (μPV) values were significantly larger than the N _2_-derived BET specific surface area (S _BET_) and pore volume (TPV) values signifying that kinetic limitations with N _2_ physisorption were present for all biochars and there is some degree of microporosity present.

**Figure 8.**
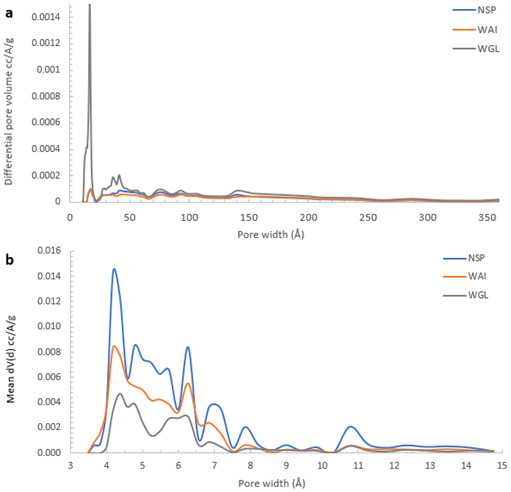
Pore volume weighted pore size-distribution derived from **a**) N _2_ (mesopore region 20–500Å) and **b**) CO _2_ (micropore region<20Å) for NSP, WAI and WGL biochars.

NSP BC showed the largest surface area measured with CO _2_ and the lowest with N _2_ indicating a more microporous structure whereas WGL BC had the highest N _2_ SSA and lowest CO _2_ µSSA signifying a slightly less microporous and more mesoporous structure. The greatest pore size distribution at pore diameters 4–15Å was recorded for NSP_BC also indicating it had more of a microporous structure than WGL_BC which recorded relatively sparse pore size distributions in this region ( Figure 8b). In the mesoporous region, (16–150 Å), WGL_BC pore size distributions were much greater than both NSP_BC and WAI_BC confirming WGL_BC has a more mesoporous structure ( Figure 8a).

The values obtained demonstrate the complex pore network within the biochar, even though the surface area values are generally low compared to other biochars there is still a degree of both microporosity and mesoporosity within the biochars. Low surface area biochars may be unsuitable for use as soil amendments as the water holding capacity is relatively low and the low porosities are not conducive to promoting soil microbial growth ( Ishii & Kadoya, 1994; Thies & Rillig, 2009), which play an important role in nutrient cycling ( Lambers *et al.*, 2008). The surface area could be increased by increasing the pyrolysis temperature ( Song & Guo, 2012; Tomczyk *et al.*, 2020). The fast pyrolysis of municipal sludge biochar at temperatures 500 – 900 °C showed that increasing temperatures resulted in a greater microporous network within the biochar ( Chen *et al.*, 2014). Previous work has shown that the greatest enhancement of sewage sludge biochar porosity occurred between 400 – 600°C ( Bagreev *et al.*, 2001). However heavy metal concentration in biochars generally increase with pyrolytic temperature ( Lu *et al.*, 2013). This is because heavy metals do not volatilize, so their concentration within the biochar increases with pyrolysis temperature ( Chanaka Udayanga *et al.*, 2019; Hossain *et al.*, 2011; Wang *et al.*, 2021).

The larger surface areas (with CO _2_) and more microporous structure of NSP_BC and WAI_BC relates to their lower ash content of 67.0% and 62.3% respectively. The lowest surface area measured (with CO _2_) was that of WGL_BC which recorded the highest ash content of 88.3%. These findings support the notion that lower surface areas relate to ash filling or blocking access to the biochar micropores ( Song & Guo, 2012).

### Zeta potential

Zeta (electrokinetic) potential signifies the net charge between the surface plane and slip plane of a colloidal particle ( Hiemenz & Rajagopalan, 1997). Zeta potential values yield information about the external surface charges of biochar particles in solution and indicates the sorption and nutrient holding characteristics of the biochar in soil. Negatively charged surfaces are unlikely to sorb negatively charged ions such as phosphate but are more likely to sorb positive cations such as heavy metal ions and ammonium ions.

**Figure 9.**
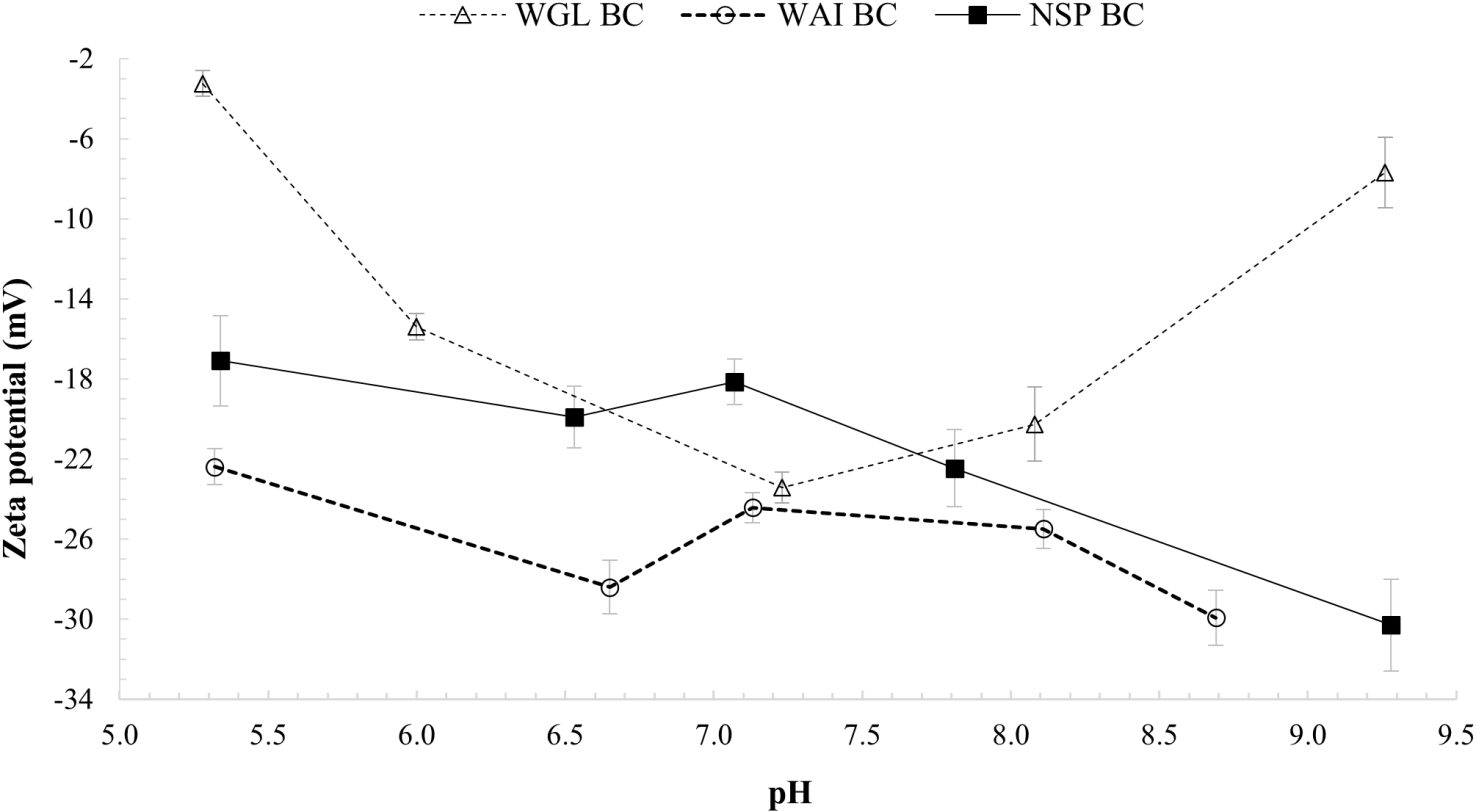
Zeta potentials of WGL_BC, WAI_BC and NSP_BC at pH values from 5-9.5.

The zeta potential values for all three biochar samples were negative in the pH range 5.0–9.5, revealing that negative charges are carried on the surface of the biochar particles ( Figure 9). FTIR spectra revealed the existence of oxygen containing functional groups (–COO ^−^ and–OH) on the biochars surface which can contribute considerably to surface charge of the biochars. The negative zeta potentials of all three biochars in the pH range 5.0 – 9.5 support this interpretation.

At acidic pH, the zeta potentials of the biochar samples became less negative, indicating that the association of –COO ^−^ and –O ^−^ with H ^+^ reduced the negative charge of the biochars. With increasing pH, the zeta potential of WAI_BC and NSP_BC biochars become more negative due to increasing deprotonation of the biochar surface functional groups ( Yuan *et al.*, 2011). However, at pH above 7 there was an increase in zeta potential for WGL_BC from -23.4mV to -7.7mV indicating a decrease in negative surface charge. WGL_BC contains the highest ash content of all the biochars ( Table 1), and it is likely this that contributes to the increase in zeta potential values at pH>7. The mechanism by which surface charge increases at high pH values cannot be explained by deprotonation of the surface functional groups in the case of WGL_BC. The higher ash content indicates some other mechanism occurring. Zeta potential of fine coal tailings containing several ash-forming minerals showed a similar trend which the authors attributed to the presence of alumina and silicate particles, which result in lower negative zeta potential values. They also noted that varying zeta potential values at high pH could be attributed to the binding of more cations such as Ca ^2+ ^( Kumar *et al.*, 2014). Positively charged calcium monohydroxide ions on the biochar surface would to some degree neutralize the negative surface charges resulting in less negative zeta potential values. ( Liu *et al.*, 2002). It is possible that at pH values >9 WGL_BC would have positive zeta potential values and thus more likely to sorb negatively charged ions such as nitrate or phosphate. 

### X-ray diffraction (XRD)

**Figure 10.**
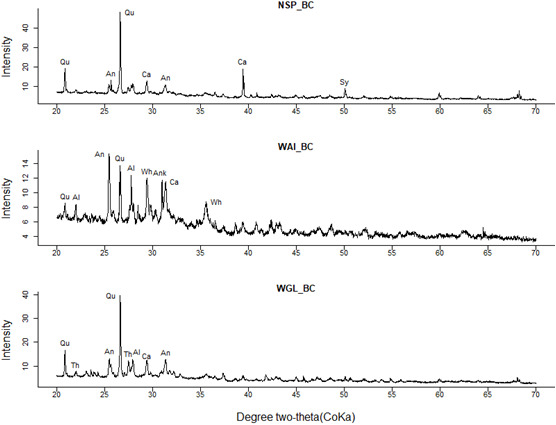
XRD patterns of NSP, WAI and WGL biochar (Qu= Quartz, Al=Albite, Ca=Calcite, An = Anhydrite, Sy = Sylvite, Wh = Whewellite, Ank=Ankerite, Th= Thermonatrite).

X-ray diffraction (XRD) analysis of the biochars revealed that mineral components in the crystal form were present in all three biochars ( Figure 10). Quartz was identified as the predominant crystalline phase with the highest peak at 2θ around 26.6° ( *d* = 3.33 Å) in NSP and WGL biochars. WAI biochar exhibited a more intense peak relating to CaSO _4_ (anhydrite). Quartz, sylvite, calcite, calcium sulphate, albite were the most common phases identified. These minerals are formed during pyrolysis due to a reaction between CO _2_ and alkaline-earth metals and alkaline oxyhydroxides.

Previous research has shown sewage sludge biochar to have a turbostratic structure where the carbon fraction is dominated by disordered graphitic crystallites ( Srinivasan *et al.*, 2015; Uchimiya *et al.*, 2011). This is in discordance with the XRD results which show a lack of C (002) diffraction peaks (2θ = 15-30°) and C (101) diffraction peaks (2θ = 40-50°) due to amorphous carbon structures and graphite structures respectively. The biochars studied here do have a very high ash content and the lack of these peaks could be as a result of interference of high-intensity quartz peaks. Studies have shown that the high content of minerals, specifically quartz can affect the structural characterization of biochar carbon fraction ( Feng *et al.*, 2015).

The difference in mineral composition between the three biochars could be due to possible contamination of FS by sand and grit caused by poorly lined containment structures ( Niwagaba *et al.*, 2014). The containment structures at each location would have to be investigated to reach a definitive conclusion. Overall, the mineralogical composition of the biochars is in agreement with their high ash contents.

### Scanning electron microscopy (SEM) with energy dispersive x-ray analysis (EDX)

**Figure 11.**
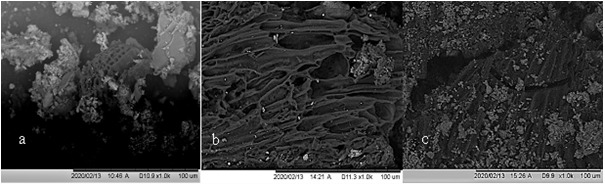
SEM micrograph of ( **a**) Original WGL biochar, ( **b**) Original NSP biochar, ( **c**) Original WAI biochar.

**Figure 12.**
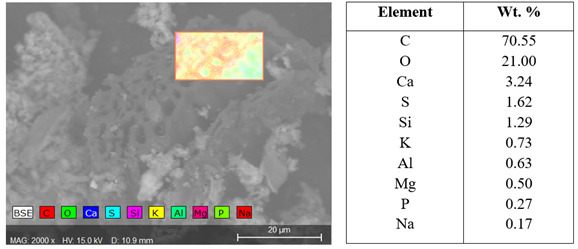
SEM-EDX map for all elements distribution across the area highlighted in image and associated energy dispersive X-ray (EDX) quantification of biochar.

SEM analysis reveals a complex porous structure evident in all biochars ( Figure 11). The porous structure of biochars strongly resembles the cellular structure of the original feedstock ( Fuertes *et al.*, 2010; Yao *et al.*, 2011). In the case of faecal sludge, cellular macroporous structures arise from undigested fibrous vegetable matter. The morphology of the biochar is honeycomb-like with cylindrical and slit like holes clearly observable. This porous structure can provide a specialized environment for the colonization of microbes ( Thies & Rillig, 2012). This increase in mycorrhizal fungi contributes to increased mineralization of recalcitrant soil organic matter, ultimately improving soil and plant health ( Anderson *et al.*, 2011; Zimmerman *et al.*, 2011). SEM images also show all three biochars have high ash content with EDX confirming the presence of mineral elements ( Figure 12). The SEM images clearly showed a high presence of clay mineral particles/ash (white/grey) with a smaller amount of biochar particles present (black).

Visually WGL biochar had a higher ash content which is concurrent with the ash percentage from proximate analyses. EDX results on the biochar particles themselves revealed high volumes of carbon and oxygen ( Figure 12). Also present were silicon, calcium aluminium, potassium, magnesium, phosphorus, and sodium all of which are beneficial to plant health.

### Cation exchange capacity

Cation exchange capacity (CEC) enables biochars to adsorb cationic nutrients such as NH _4_^+^, Ca ^2+^, and K ^+^. It is thought this characteristic of biochar results predominantly from formation of carboxylic functional groups during oxidation ( Cheng *et al.*, 2006).

There was a large variation in CEC values with WGL biochar (WGL BC) the highest CEC at 129.3 cmolKg ^-1^ and NSP biochar the lowest CEC at 41.9 cmolKg ^-1^ ( Table 1). Fresh biochars from lignocellulosic biomass generally have lower CEC, with manure-based biochars exhibiting higher CEC values ( Tag *et al.*, 2016). In the literature CEC values for biochar are highly variable, commonly ranging from 6 cmol _(+)_ Kg ^−1^ ( Munera-Echeverri *et al.*, 2018) to 36.3 cmol _(+)_ Kg ^−1^ ( Song & Guo, 2012) to as high as 304 cmol _(+)_ Kg ^−1^.

Yuan *et al.* (2011) proposed that high ash content biomass creates high CEC biochars and that K, Na, Ca, Mg, and P in the feedstock would promote formation of O-containing acidic functional groups such as carboxylic, and phenolic groups on biochar surface during pyrolysis and thus, result in higher CEC ( Gaskin *et al.*, 2008). However, FTIR analysis showed a lack of acidic functional groups such as phenolic groups in these biochars. It is possible that the high ash content of these biochars could contribute to methodological problems in determining CEC ( Graber *et al.*, 2017). There is a large range of CEC values reported in the literature and measurements are often poorly reproducible ( Munera-Echeverri *et al.*, 2018). FTIR shows that there are carbonates and silicates present in these biochars which would result in the release of base cations and interference with the sum of measured exchangeable base cations ( Munera-Echeverri *et al.*, 2018). WGL biochar records the highest CEC value (129.3 ± 2.3 cmol.kg ^-1^) and the highest ash content of all three biochars implying that it is the high ash content that is responsible for the high CEC value.

## Conclusion

Overall, all three faecal sludge biochars had a high ash content, high pH, low carbon content, negative surface charge and low specific surface areas and pore volumes. The similarity of FTIR spectra between biochars signifies a uniformity of the organic component of all three biochars. Warangal biochar had a significantly higher ash content and pH compared to the Narsapur and Wai biochar. There were also differences in XRD spectra between biochars. These differences are likely related to the contamination of faecal sludge in the containment structure by sand or grit, or the sintered mineral deposits in the reactor itself. The variability of these faecal sludge biochar properties highlights the differences between small-scall laboratory and full-scale “real world” biochar production. Control over every single variable in large-scale faecal sludge biochar production is difficult and routine inspections of every containment structure at every location would be time-consuming. However, the pH and ash content of the biochars could be monitored periodically at the treatment plant. The low surface areas and porosity of these biochars could prove detrimental in its end use a soil amendment as these properties relate to water holding capacity and microbial activity. However, increasing the porosity of faecal sludge biochar is possible through techniques such as chemical and physical activation. Overall, the properties of these biochars, in particular the high alkalinity, shows their potential use as soil amendment particularly with acidic soil. The liming effect from these biochars and release of nutrients from the ash in the biochar itself could contribute to increased agricultural productivity especially in developing nations where the use of inorganic fertilizer on smallholder farmers is much lower. Future work should determine the biochars total and plant available macro-and micronutrient concentrations. Further investigation into the evaluation of these biochars as soil amendments with a focus on application to acidic soils is also recommended.

## Data Availability

Mendeley Data: FS biochar properties. https://doi.org/10.17632/2xsdbdb38k.3 (Nicholas, 2022) This project contains the following files: Figure 3 FTIR original_final600.tiff- FTIR spectra unamended biochars Figure 4 FTIR ashed_final600.tiff – FTIR spectra of ashed biochars Figure 5 FTIR acidwashed_final600.tiff – FTIR spectra of acid washed biochars Figure 6 Nitrogen.tif – N _2_ adsorption and desorption isotherms of WAI, WGL and NSP biochars Figure 7 CO2.tif – CO _2_ adsorption and desorption isotherms of WAI, WGL and NSP biochars Figure 8 pore volumes600.tif - Pore volume weighted pore size-distribution graphs for a) N _2_ (mesopore region 2-50nm) and b) CO _2_ (micropore region<2nm) for NSP, WAI and WGL biochars. Figure 9 zeta potential600.tif – Zeta potentials of WGL_BC, WAI_BC and NSP_BC at pH values from 5-9.5 Figure 10 XRD500.tif *-* XRD spectra of NSP, WAI and WGL biochar Figure 11 SEM500.tif- SEM biochar images Figure 12 EDX500.tif – EDX biochar image CEC2.xlsx – All cation exchange capacity data CHNS_results.xlsx – Elemental analysis (CHNS) data FTIR Data2.xlsx – all FTIR data XRD15.xlsx – Xray diffraction data N2 isotherm data.xlsx – Nitrogen adsorption and desorption isotherms data N2 Pore volume BET data.xlsx – Nitrogen pore volume data CO2 isotherm data.xlsx – Carbon dioxide adsorption and desorption isotherms data CO2 BET pore volume data.xlsx – Carbon dioxide pore volume data ph and electrical conductivity raw data.xlsx – pH and electrical conductivity raw data Raw zeta potential data.xlsx – raw zeta potential data Data are available under the terms of the Creative Commons Attribution 4.0 International license (CC-BY 4.0).

## References

[ref-1] Agrafioti E Bouras G Kalderis D : Biochar production by sewage sludge pyrolysis. *J Anal Appl Pyrolysis.* 2013;101:72–78. 10.1016/j.jaap.2013.02.010

[ref-112] Ahmad M Rajapaksha AU Lim JE : Biochar as a sorbent for contaminant management in soil and water: A review. *Chemosphere.* 2014;99:19–33. 10.1016/j.chemosphere.2013.10.071 24289982

[ref-2] Anderson CR Condron LM Clough TJ : Biochar induced soil microbial community change: Implications for biogeochemical cycling of carbon, nitrogen and phosphorus. *Pedobiologia (Jena).* 2011;54(5–6):309–320. 10.1016/j.pedobi.2011.07.005

[ref-3] Andriessen N Ward BJ Strande L : To char or not to char? Review of technologies to produce solid fuels for resource recovery from faecal sludge. *J Water Sanit Hyg Dev.* 2019;9(2):210–224. 10.2166/washdev.2019.184

[ref-4] ASTM D 1762-84: Standard Test Method for Chemical Analysis of Wood Charcoal. *ASTM Int.* 2011;84:1–2. Reference Source

[ref-113] Awad YM Ok YS Abrigata J : Pine sawdust biomass and biochars at different pyrolysis temperatures change soil redox processes. *Sci Total Environ.* 2018;625:147–154. 10.1016/j.scitotenv.2017.12.194 29289000

[ref-114] Awere E Appiah Obeng P Asirifua Obeng P : Characterization of Faecal Sludge from Pit Latrines to Guide Management Solutions in Cape Coast, Ghana. *J Geogr Environ Earth Sci Int.* 2020;24(1):1–13. 10.9734/jgeesi/2020/v24i130189

[ref-5] Bagreev A Bandosz TJ Locke DC : Pore structure and surface chemistry of adsorbents obtained by pyrolysis of sewage sludge-derived fertilizer. *Carbon NY.* 2001;39(13):1971–1979. 10.1016/S0008-6223(01)00026-4

[ref-115] Bai X Li Z Zhang Y : Recovery of Ammonium in Urine by Biochar Derived from Faecal Sludge and its Application as Soil Conditioner. *Waste Biomass Valor.* 2018;9:1619–1628. 10.1007/s12649-017-9906-0

[ref-6] Banik C Lawrinenko M Bakshi S : Impact of Pyrolysis Temperature and Feedstock on Surface Charge and Functional Group Chemistry of Biochars. *J Environ Qual.* 2018;47(3):452–461. 10.2134/jeq2017.11.0432 29864182

[ref-116] Barani V Hegarty-Craver M Rosario P : Characterization of fecal sludge as biomass feedstock in the southern Indian state of Tamil Nadu [version 1; peer review: 1 approved, 1 approved with reservations]. *Gates Open Res.* 2018;2:52. 10.12688/gatesopenres.12870.1 32803126 PMC7383100

[ref-117] Bassan M Tchonda T Yiougo L : Characterization of faecal sludge during dry and rainy seasons in Ouagadougou, Burkina Faso. *36th WEDC Int Conf Deliv Water Sanit Hyg Serv an Uncertain Environ*.2013;1–6. Reference Source

[ref-7] Bleuler M Gold M Strande L : Pyrolysis of Dry Toilet Substrate as a Means of Nutrient Recycling in Agricultural Systems: Potential Risks and Benefits. *Waste Biomass Valor.* 2021;12:4171–4183. 10.1007/s12649-020-01220-0

[ref-8] Brunauer S Emmett PH Teller E : Adsorption of Gases in Multimolecular Layers. *J Am Chem Soc.* 1938;60(2):309–319. 10.1021/ja01269a023

[ref-200] Calderón FJ McCarty GW Reeves JB : Pyrolisis-MS and FT-IR analysis of fresh and decomposed dairy manure. *J Anal Appl Pyrolysis.* 2006;76(1–2):14–23. 10.1016/j.jaap.2005.06.009

[ref-10] Castan S Sigmund G Hüffer T : Biochar particle aggregation in soil pore water: The influence of ionic strength and interactions with pyrene. *Environ Sci Process Impacts.* 2019;21(10):1722–1728. 10.1039/c9em00277d 31433415

[ref-11] Chan KY Van Zwieten L Meszaros I : Agronomic values of greenwaste biochar as a soil amendment. *Aust J Soil Res.* 2007;45(8):629–634. 10.1071/SR07109

[ref-12] Chanaka Udayanga WD Veksha A Giannis A : Insights into the speciation of heavy metals during pyrolysis of industrial sludge. *Sci Total Environ.* 2019;691:232–242. 10.1016/j.scitotenv.2019.07.095 31323569

[ref-14] Chen T Zhang Y Wang H : Influence of pyrolysis temperature on characteristics and heavy metal adsorptive performance of biochar derived from municipal sewage sludge. *Bioresour Technol.* 2014;164:47–54. 10.1016/j.biortech.2014.04.048 24835918

[ref-13] Chen B Zhou D Zhu L : Transitional adsorption and partition of nonpolar and polar aromatic contaminants by biochars of pine needles with different pyrolytic temperatures. *Environ Sci Technol.* 2008;42(14):5137–5143. 10.1021/es8002684 18754360

[ref-16] Cheng CH Lehmann J Thies JE : Oxidation of black carbon by biotic and abiotic processes. *Org Geochem.* 2006;37(11):1477–1488. 10.1016/j.orggeochem.2006.06.022

[ref-17] Claoston N Samsuri AW Ahmad Husni MH : Effects of pyrolysis temperature on the physicochemical properties of empty fruit bunch and rice husk biochars. *Waste Manag Res.* 2014;32(4):331–339. 10.1177/0734242X14525822 24643171

[ref-18] Coates J : Encyclopedia of Analytical Chemistry -Interpretation of Infrared Spectra, A Practical Approach. *Encycl Anal Chem.* 2004;1–23.

[ref-19] Crombie K Mašek O Cross A : Biochar - synergies and trade-offs between soil enhancing properties and C sequestration potential. *GCB Bioenergy.* 2015;7(5):1161–1175. 10.1111/gcbb.12213

[ref-20] Crombie K Mašek O Sohi SP : The effect of pyrolysis conditions on biochar stability as determined by three methods. *GCB Bioenergy.* 2013;5(2):122–131. 10.1111/gcbb.12030

[ref-21] Davis WM Erickson CL Johnston CT : Quantitative Fourier Transform Infrared spectroscopic investigation humic substance functional group composition. *Chemosphere.* 1999;38(12):2913–2928. 10.1016/S0045-6535(98)00486-X

[ref-22] Deacon GB Phillips RJ : Relationships between the carbon-oxygen stretching frequencies of carboxylato complexes and the type of carboxylate coordination. *Coord Chem Rev.* 1980;33(3):227–250. 10.1016/S0010-8545(00)80455-5

[ref-23] Downs RT Hall-Wallace M : The American Mineralogist crystal structure database. *Am Mineral.* 2003;88:247–250. 10.5860/choice.43sup-0302

[ref-24] EEC: 91/692/EEC Council Directive of 12 June 1986 on the Protection of the Environment, and in Particular of the Soil, when Sewage Sludge Is Used in Agriculture. EEC.1986.

[ref-25] Ellerbrock RH Gerke HH : FTIR spectral band shifts explained by OM-cation interactions. *J Plant Nutr Soil Sci.* 2021;184(3):388–397. 10.1002/jpln.202100056

[ref-26] Enders A Hanley K Whitman T : Characterization of biochars to evaluate recalcitrance and agronomic performance. *Bioresour Technol.* 2012;114:644–653. 10.1016/j.biortech.2012.03.022 22483559

[ref-27] Enders A Lehmann J : Proximate analyses for characterising biochars. *Biochar A Guid to Anal Methods.* 2015;9–22.

[ref-28] European Biochar Foundation: Guidelines for a Sustainable Production of Biochar. *Eur Biochar Found.* 2016;1–22.

[ref-29] Falaras P : Cottonseed Oil Bleaching by Acid-Activated Montmorillonite. *Clay Miner.* 1999;34(2):221–232. 10.1180/000985599546181

[ref-118] Fanyin-Martin A Tamakloe W Antwi E : Chemical characterization of faecal sludge in the Kumasi metropolis, Ghana [version 1; peer review: 1 approved, 1 approved with reservations]. *Gates Open Res.* 2017;1:12. 10.12688/gatesopenres.12757.1

[ref-30] Farmer VC : Chapter 1 Vibrational Spectroscopy in Mineral Chemistry.1974;1–10. 10.1180/mono-4.1

[ref-119] Farrell M Kuhn TK Macdonald LM : Microbial utilisation of biochar-derived carbon. *Sci Total Environ.* 2013;465:288–297. 10.1016/j.scitotenv.2013.03.090 23623696

[ref-31] Feng H Zheng M Dong H : Three-dimensional honeycomb-like hierarchically structured carbon for high-performance supercapacitors derived from high-ash-content sewage sludge. *J Mater Chem A.* 2015;3(29):15225–15234. 10.1039/C5TA03217B

[ref-32] Fuertes AB Arbestain MC Sevilla M : Chemical and structural properties of carbonaceous products obtained by pyrolysis and hydrothermal carbonisation of corn stover. *Aust J Soil Res.* 2010;48(7):618–626. 10.1071/SR10010

[ref-34] Gaskin JW Speir A Morris LM : Potential for Pyrolysis Char to Affect Soil Moisture and Nutrient Status of a Loamy Sand Soil.2007. Reference Source

[ref-33] Gaskin JW Steiner C Harris K : Effect of Low-Temperature Pyrolysis Conditions on Biochar for Agricultural Use. *Trans ASABE.* 2008;51(6):2061–2069. 10.13031/2013.25409

[ref-35] Glaser B Haumaier L Guggenberger G : The 'Terra Preta' phenomenon: A model for sustainable agriculture in the humid tropics. *Naturwissenschaften.* 2001;88(1):37–41. 10.1007/s001140000193 11302125

[ref-36] Gold M Cunningham M Bleuler M : Operating parameters for three resource recovery options from slow-pyrolysis of faecal sludge. *J Water Sanit Hyg Dev.* 2018;8(4):707–717. 10.2166/washdev.2018.009

[ref-37] Graber E Singh B Lehmann J : Determination of Cation Exchange Capacity in Biochar. In: Singh, B., Camps-Arbestain, M., Lehmann, J. (Eds.), *Biochar: A Guide to Analytical Methods*. Csiro Publishing,2017;74–84.

[ref-38] Gražulis S Chateigner D Downs RT : Crystallography Open Database - An open-access collection of crystal structures. *J Appl Crystallogr.* 2009;42(Pt 4):726–729. 10.1107/S0021889809016690 22477773 PMC3253730

[ref-39] Gražulis S Daškevič A Merkys A : Crystallography Open Database (COD): An open-access collection of crystal structures and platform for world-wide collaboration. *Nucleic Acids Res.* 2012;40(Database issue):420–427. 10.1093/nar/gkr900 22070882 PMC3245043

[ref-40] Gražulis S Merkys A Vaitkus A : Computing stoichiometric molecular composition from crystal structures. *J Appl Crystallogr.* 2015;48(Pt 1):85–91. 10.1107/S1600576714025904 26089747 PMC4453171

[ref-41] Gwenzi W Munondo R : Long-term impacts of pasture irrigation with treated sewage effluent on nutrient status of a sandy soil in Zimbabwe. *Nutr Cycl Agroecosystems.* 2008;82:197–207. 10.1007/s10705-008-9181-3

[ref-42] Haller L Hutton G Bartram J : Estimating the costs and health benefits of water and sanitation improvements at global level. *J Water Health.* 2007;5(4):467–480. 10.2166/wh.2007.008 17878561

[ref-43] Hiemenz PC Rajagopalan R : Principles of Colloid and Surface Chemistry, Revised and Expanded. 3rd ed. CRC Press.1997. 10.1201/9781315274287

[ref-44] Hossain MK Strezov V Chan KY : Influence of pyrolysis temperature on production and nutrient properties of wastewater sludge biochar. *J Environ Manage.* 2011;92(1):223–8. 10.1016/j.jenvman.2010.09.008 20870338

[ref-120] Hossain MK Strezov V Yin Chan K : Agronomic properties of wastewater sludge biochar and bioavailability of metals in production of cherry tomato ( *Lycopersicon esculentum*). *Chemosphere.* 2010;78(9):1167–1171. 10.1016/j.chemosphere.2010.01.009 20110103

[ref-46] Hutton G Bartram J : Global costs of attaining the Millennium Development Goal for water supply and sanitation. *Bull World Health Organ.* 2008;86(1):13–19. 10.2471/blt.07.046045 18235885 PMC2647341

[ref-47] IBI: Standardized Product Definition and Product Testing Guidelines for Biochar 7 That Is Used in Soil. [WWW Document].2015; (accessed 11.10.20). Reference Source

[ref-48] Ishii T Kadoya K : Effects of charcoal as a soil conditioner on citrus growth and vesicular-arbuscular mycorrhizal development. *J Japanese Soc Hortic Sci.* 1994;63(3):529–535. 10.2503/jjshs.63.529

[ref-49] Jeffery S Verheijen FGA van der Velde M : A quantitative review of the effects of biochar application to soils on crop productivity using meta-analysis. *Agric Ecosyst Environ.* 2011;144(1):175–187. 10.1016/j.agee.2011.08.015

[ref-50] Jiang W Saxena A Song B : Elucidation of functional groups on gram-positive and gram-negative bacterial surfaces using infrared spectroscopy. *Langmuir.* 2004;20(26):11433–11442. 10.1021/la049043+ 15595767

[ref-51] Keiluweit M Nico PS Johnson MG : Dynamic molecular structure of plant biomass-derived black carbon (biochar). *Environ Sci Technol.* 2010;44(4):1247–1253. 10.1021/es9031419 20099810

[ref-52] Khan S Chao C Waqas M : Sewage sludge biochar influence upon rice ( *Oryza sativa* L) yield, metal bioaccumulation and greenhouse gas emissions from acidic paddy soil. *Environ Sci Technol.* 2013;47(15):8624–8632. 10.1021/es400554x 23796060

[ref-53] Kim P Johnson A Edmunds CW : Surface functionality and carbon structures in lignocellulosic-derived biochars produced by fast pyrolysis. *Energy and Fuels.* 2011;25(10):4693–4703. 10.1021/ef200915s

[ref-54] Klasson KT Lima IM Boihem LL : Poultry manure as raw material for mercury adsorbents in gas applications. *J Appl Poult Res.* 2009;18(3):562–569. 10.3382/japr.2009-00011

[ref-55] Koetlisi KA Muchaonyerwa P : Biochar Types from Latrine Waste and Sewage Sludge Differ in Physico-Chemical Properties and Cadmium Adsorption. *Am J Appl Sci.* 2017;14(11):1039–1048. 10.3844/ajassp.2017.1039.1048

[ref-56] Kosek M Bern C Guerrant RL : The global burden of diarrhoeal disease, as estimated from studies published between 1992 and 2000. *Bull World Health Organ.* 2003;81(3):197–204. 12764516 PMC2572419

[ref-57] Krueger BC Fowler GD Templeton MR : Resource recovery and biochar characteristics from full-scale faecal sludge treatment and co-treatment with agricultural waste. *Water Res.* 2020;169:115253. 10.1016/j.watres.2019.115253 31707178 PMC6961206

[ref-58] Kumar S Bhattacharya S Mandre NR : Characterization and flocculation studies of fine coal tailings. *J S Afr Inst Min Metall.* 2014;114(11):945–949. Reference Source

[ref-121] Lama SL Samal M Luitel S : CHARACTERIZATION OF FAECAL SLUDGE AND DESIGN OF FAECAL SLUDGE TREATMENT PLANT IN DHULIKHEL MUNICIPALITY. In: *Sanitation for All by 2030. Every Year, the World Water Day Highlights a Specific Aspect of Freshwater*.2022;17.

[ref-59] Lambers H Chapin FS Pons TL : Plant physiological ecology.Second edition.2008;1–604. 10.1007/978-0-387-78341-3

[ref-60] Lehmann J Joseph S : Biochar for Environmental Management.2012.

[ref-122] Lehmann J Rillig MC Thies J : Biochar effects on soil biota - A review. *Soil Biol Biochem.* 2011;43(9):1812–1836. 10.1016/j.soilbio.2011.04.022

[ref-61] Lima I Klasson KT Uchimiya M : Selective release of inorganic constituents in broiler manure biochars under different post-activation treatments. *J Residuals Sci Technol.* 2016;13(1):37–48. Reference Source

[ref-63] Liu X Li Z Zhang Y : Characterization of human manure-derived biochar and energy-balance analysis of slow pyrolysis process. *Waste Manag.* 2014;34(9):1619–1626. 10.1016/j.wasman.2014.05.027 24961565

[ref-62] Liu J Zhou Z Xu Z : Electrokinetic study of hexane droplets in surfactant solutions and process water of bitumen extraction systems. *Ind Eng Chem Res.* 2002;41(1):52–57. 10.1021/ie010543x

[ref-64] Lou L Luo L Wang L : The influence of acid demineralization on surface characteristics of black carbon and its sorption for pentachlorophenol. *J Colloid Interface Sci.* 2011;361(1):226–231. 10.1016/j.jcis.2011.05.015 21658703

[ref-65] Lu H Zhang W Wang S : Characterization of sewage sludge-derived biochars from different feedstocks and pyrolysis temperatures. *J Anal Appl Pyrolysis.* 2013;102:137–143. 10.1016/j.jaap.2013.03.004

[ref-66] Mara D Lane J Scott B : Sanitation and health. *PLoS Med.* 2010;7(11):e1000363. 10.1371/journal.pmed.1000363 21125018 PMC2981586

[ref-123] Méndez A Gómez A Paz-Ferreiro J : Effects of sewage sludge biochar on plant metal availability after application to a Mediterranean soil. *Chemosphere.* 2012;89(11):1354–1359. 10.1016/j.chemosphere.2012.05.092 22732302

[ref-67] Merkys A Vaitkus A Butkus J : *COD::CIF::Parser*: An error-correcting CIF parser for the Perl language. *J Appl Crystallogr.* 2016;49(Pt 1):292–301. 10.1107/S1600576715022396 26937241 PMC4762566

[ref-68] Mokaya R Jones W : Pillared clays and pillared acid-activated clays: A comparative-study of physical, acidic, and catalytic properties. *J Catal.* 1995;153(1):76–85. 10.1006/jcat.1995.1109

[ref-69] Munera-Echeverri JL Martinsen V Strand LT : Cation exchange capacity of biochar: An urgent method modification. *Sci Total Environ.* 2018;642:190–197. 10.1016/j.scitotenv.2018.06.017 29894878

[ref-71] Niwagaba CB Mbéguéré M Strande L : Faecal sludge quantification, characterisation and treatment objectives.In: Linda Strande, Mariska Ronteltap, D.B. (Editor) (Ed.), *Faecal Sludge Management: Systems Approach for Implementation and Operation.* IWA Publishing, London, UK,2014;19–44. Reference Source

[ref-72] Novak JM Busscher WJ Laird DL : Impact of biochar amendment on fertility of a southeastern coastal plain soil. *Soil Sci.* 2009;174(2):105–112. 10.1097/SS.0b013e3181981d9a

[ref-124] Park JH Choppala GK Bolan NS : Biochar reduces the bioavailability and phytotoxicity of heavy metals. *Plant Soil.* 2011;348:439–451. 10.1007/s11104-011-0948-y

[ref-73] Peech M Alexander LT Dean LA : Methods of Soil Analysis for Soil Fertility Investigations.Washington, DC,1947. Reference Source

[ref-74] Qian L Chen B : Dual role of biochars as adsorbents for aluminum: The effects of oxygen-containing organic components and the scattering of silicate particles. *Environ Sci Technol.* 2013;47(15):8759–8768. 10.1021/es401756h 23826729

[ref-75] Quirós M Gražulis S Girdzijauskaitė S : Using SMILES strings for the description of chemical connectivity in the Crystallography Open Database. *J Cheminform.* 2018;10(1):23. 10.1186/s13321-018-0279-6 29777317 PMC5959826

[ref-76] Rehrah D Reddy MR Novak JM : Production and characterization of biochars from agricultural by-products for use in soil quality enhancement. *J Anal Appl Pyrolysis.* 2014;108:301–309. 10.1016/j.jaap.2014.03.008

[ref-77] Samsuri AW Sadegh-Zadeh F Seh-Bardan BJ : Adsorption of As(III) and As(V) by Fe coated biochars and biochars produced from empty fruit bunch and rice husk. *J Environ Chem Eng.* 2013;1(4):981–988. 10.1016/j.jece.2013.08.009

[ref-78] Schimmelpfennig S Glaser B : One Step Forward toward Characterization: Some Important Material Properties to Distinguish Biochars. *J Environ Qual.* 2012;41(4):1001–1013. 10.2134/jeq2011.0146 22751042

[ref-125] Schoebitz L Bassan M Ferré A : Conference paper: FAQ: faecal sludge quantification and characterization - field trial of methodology in Hanoi, Vietnam. 37th WEDC Int Conf.2014;1–6. Reference Source

[ref-126] Septien S Mirara SW Makununika BSN : Effect of drying on the physical and chemical properties of faecal sludge for its reuse. *J Environ Chem Eng.* 2020;8(1):103652. 10.1016/j.jece.2019.103652 32140408 PMC7043394

[ref-79] Shahrokh Abadi MH Delbari A Fakoor Z : Effects of annealing temperature on infrared spectra of SiO _2_ extracted from rice husk. *J Ceram Sci Technol.* 2015;6(1):41–45. 10.4416/JCST2014-00028

[ref-80] Sharma RK Wooten JB Baliga VL : Characterization of chars from pyrolysis of lignin. *Fuel.* 2004;83(11–12):1469–1482. 10.1016/j.fuel.2003.11.015

[ref-81] Sing KSW Williams RT : Physisorption hysteresis loops and the characterization of nanoporous materials. *Adsorpt Sci Technol.* 2004;22(10):773–782. 10.1260/0263617053499032

[ref-82] Singh B Dolk MM Shen Q : Biochar pH, electrical conductivity and liming potential. *Biochar A Guid to Anal Methods.* 2017;23–38. Reference Source

[ref-83] Singh B Singh BP Cowie AL : Characterisation and evaluation of biochars for their application as a soil amendment. *Aust J Soil Res.* 2010;48(7):516–525. 10.1071/SR10058

[ref-84] Smider B Singh B : Agronomic performance of a high ash biochar in two contrasting soils. *Agric Ecosyst Environ.* 2014;191:99–107. 10.1016/j.agee.2014.01.024

[ref-85] Socrates G : Infrared and Raman Characteristic Group Frequencies, 3rd ed.John Wiley & Sons, Hoboken, NJ.2001. Reference Source

[ref-86] Song W Guo M : Quality variations of poultry litter biochar generated at different pyrolysis temperatures. *J Anal Appl Pyrolysis.* 2012;94:138–145. 10.1016/j.jaap.2011.11.018

[ref-87] Srinivasan P Sarmah AK Smernik R : A feasibility study of agricultural and sewage biomass as biochar, bioenergy and biocomposite feedstock: Production, characterization and potential applications. *Sci Total Environ.* 2015;512–513:495–505. 10.1016/j.scitotenv.2015.01.068 25644846

[ref-88] Strande L Brdjanovic D Ronteltap M : Faecal Sludge Management: Systems Approach for Implementation and Operation. IWA Publishing, London, UK.2014. 10.2166/9781780404738

[ref-89] Stuart BH : Infrared Spectroscopy: Fundamentals and Applications, Analytical Techniques in the Sciences. John Wiley & Sons, Ltd, Chichester, UK.2004. 10.1002/0470011149

[ref-90] Tag AT Duman G Ucar S : Effects of feedstock type and pyrolysis temperature on potential applications of biochar. *J Anal Appl Pyrolysis.* 2016;120:200–206. 10.1016/j.jaap.2016.05.006

[ref-91] Thies JE Rillig MC : Characteristics of biochar: biological properties. In:Lehmann, J., Joseph, S. (Eds.), *Biochar for Environmental Management*. Earthscan, Gateshead, UK,2009;85–105. 10.4324/9781849770552

[ref-92] Thies JE Rillig MC : Characteristics of biochar: Biological properties. *Biochar Environ Manag Sci Technol.* 2012;85–105.

[ref-93] Thomas Klasson K Uchimiya M Lima IM : Uncovering surface area and micropores in almond shell biochars by rainwater wash. *Chemosphere.* 2014;111:129–134. 10.1016/j.chemosphere.2014.03.065 24997909

[ref-94] Thommes M Kaneko K Neimark AV : Physisorption of gases, with special reference to the evaluation of surface area and pore size distribution (IUPAC Technical Report). *Pure Appl Chem.* 2015;87:1051–1069. 10.1515/pac-2014-1117

[ref-95] Tide Technocrats: Thermal FSSTP [WWW Document]. n.d; (accessed 7.8.22). Reference Source

[ref-96] Tomczyk A Sokołowska Z Boguta P : Biochar physicochemical properties: pyrolysis temperature and feedstock kind effects. *Rev Environ Sci Biotechnol.* 2020;19:191–215. 10.1007/s11157-020-09523-3

[ref-97] Uchimiya M Wartelle LH Klasson KT : Influence of pyrolysis temperature on biochar property and function as a heavy metal sorbent in soil. *J Agric Food Chem.* 2011;59(6):2501–2510. 10.1021/jf104206c 21348519

[ref-98] UN: Transforming our world: the 2030 Agenda for Sustainable Development. United Nations, New York [WWW Document].2015; (accessed 1.13.22). Reference Source

[ref-100] UNICEF/WHO: Progress on Household Drinking Water.2021.

[ref-99] UNICEF, WHO: Progress on Drinking Water, Sanitation and Hygiene. Joint Monitoring Programme 2017 Update and SDG Baselines. Who2017;66. Reference Source

[ref-201] von Uexküll H Mutert E : Global extent, development and economic impact of acid soils. In: *Plant-Soil Interactions at Low PH: Principles and Management*. Kluwer Academic Publishers, Dordrecht, The Netherlands,1995;5–19. 10.1007/978-94-011-0221-6_1

[ref-101] Wang Z Liu S Liu K : Effect of temperature on pyrolysis of sewage sludge: Biochar properties and environmental risks from heavy metals. *E3S Web Conf.* 2021;237.

[ref-102] Weber K Quicker P : Properties of biochar. *Fuel.* 2018;217:240–261. 10.1016/j.fuel.2017.12.054

[ref-103] Wen B Zhang JJ Zhang SZ : Phenanthrene sorption to soil humic acid and different humin fractions. *Environ Sci Technol.* 2007;41(9):3165–3171. 10.1021/es062262s 17539521

[ref-104] WHO, UNICEF: Progress on household drinking water, sanitation and hygiene 2000-2017. Special focus on inequalities. New York: United Nations Children’s Fund (UNICEF) and World Health Organization,2017;2019. Reference Source

[ref-105] Woldetsadik D Drechsel P Marschner B : Effect of biochar derived from faecal matter on yield and nutrient content of lettuce ( *Lactuca sativa*) in two contrasting soils. *Environ Syst Res.* 2018;6:2. 10.1186/s40068-017-0082-9

[ref-106] Yao Y Gao B Inyang M : Biochar derived from anaerobically digested sugar beet tailings: Characterization and phosphate removal potential. *Bioresour Technol.* 2011;102(10):6273–6278. 10.1016/j.biortech.2011.03.006 21450461

[ref-127] Yu XY Ying GG Kookana RS : Reduced plant uptake of pesticides with biochar additions to soil. *Chemosphere.* 2009;76(5):665–671. 10.1016/j.chemosphere.2009.04.001 19419749

[ref-107] Yuan JH Xu RK Zhang H : The forms of alkalis in the biochar produced from crop residues at different temperatures. *Bioresour Technol.* 2011;102(3):3488–3497. 10.1016/j.biortech.2010.11.018 21112777

[ref-108] Zhang J Lü F Zhang H : Multiscale visualization of the structural and characteristic changes of sewage sludge biochar oriented towards potential agronomic and environmental implication. *Sci Rep.* 2015;5:9406. 10.1038/srep09406 25802185 PMC4371148

[ref-128] Zhang X Wang H He L : Using biochar for remediation of soils contaminated with heavy metals and organic pollutants. *Environ Sci Pollut Res.* 2013;20(12):8472–8483. 10.1007/s11356-013-1659-0 23589248

[ref-109] Zhao L Cao X Mašek O : Heterogeneity of biochar properties as a function of feedstock sources and production temperatures. *J Hazard Mater.* 2013;256–257:1–9. 10.1016/j.jhazmat.2013.04.015 23669784

[ref-110] Zielińska A Oleszczuk P Charmas B : Effect of sewage sludge properties on the biochar characteristic. *J Anal Appl Pyrolysis.* 2015;112:201–213. 10.1016/j.jaap.2015.01.025

[ref-111] Zimmerman AR Gao B Ahn MY : Positive and negative carbon mineralization priming effects among a variety of biochar-amended soils. *Soil Biol Biochem.* 2011;43(6):1169–1179. 10.1016/j.soilbio.2011.02.005

[ref-129] Zuma L Velkushanova K Buckley C : Chemical and thermal properties of VIP latrine sludge. *Water SA.* 2015;41(4):534–540. 10.4314/wsa.v41i4.13

