# Supplementary material for: Physico-chemical properties of waste derived biochar from community scale faecal sludge treatment plants
Source: Gates Open Res. 2022 Dec 13;6:96. Originally published 2022 Aug 4. [Version 2] doi: 10.12688/gatesopenres.13727.2 (PMC10409984; doi:10.12688/gatesopenres.13727.2)
Supplement: Peer review history

# Peer review: Reviewer response for version 2

**DOI:** 10.21956/gatesopenres.15550.r34267

**Published:** 2023-08-08

**Authors:** Sadib Bin Kabir

**Affiliations:** 1 Monash University, Clayton, Victoria, Australia

This study focuses on the biochar assessment derived from faecal sludge pyrolysis. Even though numerous research on small-scale faecal sludge-derived biochar characterization has been studied before, this study conducted the characterization of biochar in a full-scale operation. The work is clearly and accurately presented and it cites the current literature.

 The study design is appropriate and the work is technically sound. This study's characterization methods include Fourier transform infrared (FTIR) spectroscopy, scanning electron microscopy (SEM), energy dispersive x-ray (EDX) spectroscopy, crystal x-ray diffraction (XRD), proximate analyses, and BET surface area porosimetry. The methods are explained in detail and are sufficient to allow replication by others. 

 Results showed that biochar samples had low specific surface area, high alkaline pH values, high ash content, and negative surface charge. In the Results section, the Fourier transform infrared section requires more improvement and critical discussion. The authors listed all the compounds present without proper explanation. X-ray diffraction analysis showed the mineral composition of each biochar differed slightly. Scanning electron microscopy analysis indicated a porous structure of each biochar with ash particles evident.

 All the source data underlying the results ensure full reproducibility. The conclusions are drawn adequately supported by the results.

Is the work clearly and accurately presented and does it cite the current literature?

Yes

If applicable, is the statistical analysis and its interpretation appropriate?

Yes

Are all the source data underlying the results available to ensure full reproducibility?

Yes

Is the study design appropriate and is the work technically sound?

Yes

Are the conclusions drawn adequately supported by the results?

Yes

Are sufficient details of methods and analysis provided to allow replication by others?

Yes

Reviewer Expertise:

Faecal Sludge Treatment, Solid waste management, Wastewater treatment, Biofuel production, Solar-thermal energy storage system assessment.

I confirm that I have read this submission and believe that I have an appropriate level of expertise to confirm that it is of an acceptable scientific standard.

# Peer review: Reviewer response for version 2

**DOI:** 10.21956/gatesopenres.15550.r34268

**Published:** 2023-08-08

**Authors:** Bhekumuzi P. Gumbi

**Affiliations:** 1 School of Chemistry and Physics, University of KwaZulu-Natal, Durban, South Africa

**Overview**

 The Authors report on very important topic of nowadays  circular economy of faecal sludge. Authors produced biochar from waste and characterized the biochar to find physico-chemical properties that can make it useful as soil enhancer. Various instrument were use to characterize the biochar including techniques that are not normal employed in the literature for characterization of biochars, which make this study significant.

**Observations**

- The authors should list/tabulates the properties of soil enhancers and comments whether this biochar fit the criteria for application in soil conditioning.
- The authors should report data with standard deviation since they mentioned analysis was done in triplicate.
- Authors must be consistent when reporting units and heading font

**Comments**

*Method Section*

- Authors must provide more information ICP: calibrations curves, validations, linearity and procedures and preparation of standard.
- Subheading (measurement of potential) between HCL and Suspension; full stop is flying.
- Authors need to provide more details on procedures for determination of CECs.

*Results and Discussion*

- Authors need to clarify whether Ash content and acidic functional groups are good or not for soil conditioning, and elaborate on how are correlated in this study. 
- The XRD pattern predict as produced biochar has high crystallinity due to high ash content. Would authors explain whether crystallinity in general is an indication of high ash content.

Is the work clearly and accurately presented and does it cite the current literature?

Yes

If applicable, is the statistical analysis and its interpretation appropriate?

Partly

Are all the source data underlying the results available to ensure full reproducibility?

Yes

Is the study design appropriate and is the work technically sound?

Yes

Are the conclusions drawn adequately supported by the results?

Yes

Are sufficient details of methods and analysis provided to allow replication by others?

Partly

Reviewer Expertise:

Environmental Chemistry, Material Science, Biochar, Analytical chemistry, chromatography, electrochemistry

I confirm that I have read this submission and believe that I have an appropriate level of expertise to confirm that it is of an acceptable scientific standard, however I have significant reservations, as outlined above.

# Peer review: Reviewer response for version 2

**DOI:** 10.21956/gatesopenres.15550.r33867

**Published:** 2023-07-06

**Authors:** Musa Manga

**Affiliations:** 1 The University of North Carolina at Chapel Hill, Chapel Hill, North Carolina, USA

**Broad overview**

 The authors discuss and compare the properties of biochar obtained from the pyrolysis of faecal sludge at three treatment plants in India. They examined properties that influence its use as soil amendment/conditioner such as the ash content, pH, carbon content, organic surface groups, surface charge, mineral content, pore volume. This is a very insightful paper as the authors investigate an understudied area; they studied the characteristics of biochar from full scale treatment plants as opposed to former studies which have examined biochar from laboratory experiments. The manuscript is well written, and the authors provided broad discussion on their findings. However, there are still some shortfalls in the manuscript (as outlined below) that the authors need to address.

**General comments **

- The authors did not state anywhere in the manuscript the reason for choosing the three different faecal sludge treatment plants (FSTPs). It will be good if the authors can clearly state why they chose these plants: are they the only three FSTPs in India? are they representative of FSTPs in India?
- Since the authors studied properties of biochar from large scale treatment plants, more value will be given to the paper if the authors can include in their discussion,  comparisons between properties of biochar obtained from treatment plants and those obtained from laboratory experiments.
- In addition, since the authors decided to study the properties of biochar for use as a soil amendment, the authors should provide a separate section where they elaborately discuss the implications of these properties on biochar’s use as soil amendment. In this section, they should also discuss what can be done to improve some of the properties that limit its use as soil amendment.  

**Specific comments**

Methods section *:*

*****Under biochar preparation* **:**

 Figure 2 was mentioned before Figure 1, this should be corrected.

*Under Characterization of biochar* **:**

 Rather than saying “The biochar characterized were collected from the pyrolizer”, it will be better to say “The following analysis were carried out on the collected biochar”

*****Under chemical analysis:*

 The full meaning of C, H, N, S should be provided at the point of first mention.****

****

*Under proximate analysis:*

 In this section, the authors state that the crucibles were first dried at a temperature of 750°C to remove moisture and other volatiles that are present. However, this temperature seems too high for just drying crucibles. The authors need to revisit this.  

Results section:

*****Under the FTIR Spectroscopy* **:**

 The authors state that the constant values obtained in the study is an “an indication of the homogenous nature of faecal sludge” However, faecal sludge cannot be said to be homogenous in nature, it is rather heterogeneous.

**Review based on Gates Open Research Format: **

- Is the work clearly and accurately presented and does it cite current literature?

The work is clearly written and cites current literature.

- Is the study design appropriate and is the work technically sound?

No study design was provided in the work, samples were collected only once from the study areas. It would have been better if the samples were collected over a period of time and not just one time sample collection.

- Are sufficient details of methods and analysis provided to allow replication by others?

The authors provided elaborate descriptions of the methods they applied in their study which enables easy replication.

- If applicable, is the statistical analysis and its interpretation appropriate?

The authors did not provide good statistical analysis. They stated that the measure of uncertainty in the study is the standard deviation. However, they did not state how many samples they used for each analysis from each plant.

- Are all the source data underlying the results available to ensure full reproducibility?

YES, the authors provided all sources of data underlying the results from the study to ensure full reproducibility.

- Are the conclusions drawn adequately supported by the results?

The conclusions drawn from the study are well supported by the results. However, more conclusions and recommendations can still be made from the results.

Is the work clearly and accurately presented and does it cite the current literature?

Yes

If applicable, is the statistical analysis and its interpretation appropriate?

Partly

Are all the source data underlying the results available to ensure full reproducibility?

Yes

Is the study design appropriate and is the work technically sound?

Partly

Are the conclusions drawn adequately supported by the results?

Yes

Are sufficient details of methods and analysis provided to allow replication by others?

Yes

Reviewer Expertise:

Public health; epidemiology;  water, sanitation and hygiene (WaSH);

I confirm that I have read this submission and believe that I have an appropriate level of expertise to confirm that it is of an acceptable scientific standard, however I have significant reservations, as outlined above.

# Peer review: Reviewer response for version 1

**DOI:** 10.21956/gatesopenres.15015.r32521

**Published:** 2022-10-06

**Authors:** Santiago Septien Stringel

**Affiliations:** 1 WASH R&D Centre (formerly known as the Pollution Research Group), University of KwaZulu-Natal, Durban, South Africa

The reviewed paper provides the characterization of biochar issued from faecal sludge from three distinct faecal sludge treatment plants in India. The studied characteristics of the biochar samples allowed to assess the suitability of the reuse of this type of sanitation product in agriculture as soil improver. This type of advanced biochar characterization has been rarely done in literature. The results are quite interesting and contribute to increasing the body of knowledge on biochar derived from faecal sludge, which is relatively poor for the moment.

 The paper is very well written in general, the results well discussed and analyzed, and the conclusion is concise and pertinent. Definitely, the paper deserves to be indexed after minor corrections.

Abstract:

- No comments.

Keywords:

- Some keywords are redundant: “faecal sludge” and “fecal”. Remove one of the two.

Introduction:

- Paragraph 6: there are more advantages of the use of biochar as soil conditioner that you can mention (e.g., removal of pollutants, reduction of pollution of underground water, support the growth of microbial communities beneficial for plant growth, retention of nutrients).
- Paragraph 6: concerning the literature about faecal sludge derived biochar, you should provide more details of what has been already studied, the objectives of the studies and the identified gaps (areas of knowledge not yet covered).

Material and Methods:

- Biochar preparation:
  - Indicate the conventional characteristics of the faecal sludge samples (i.e., moisture content, total solids, suspended solids, volatile solids, ash, COD, pH, electrical conductivity);
  - With “solar drying”, do you mean “drying beds”? Be more specific.
  - Provide more information about the pyrolyzers (estimated heating rate, throughput, residence time, carrier gas, etc...). If possible, add photographs.

 

- Characterization of biochar:
  - For all tests, indicate the number of replicates and the statistical method to measure experimental uncertainty.
  - Mention explicitly that you characterized the biochar collected from the pyrolyzer.
  - “Proximate analysis” section: for moisture content determination, be more precise on the duration of the samples in the oven.
  - “FT-IR analysis section”, paragraph 1, line 4: “infrared” must be written without capital letter
  - “Surface area section”, paragraph 2: Was the degassing done at 130°C or 105°C? It's not clear.
  - “Cation exchange capacity” section: you wrote the name of the chemical elements in full letter and by the chemical nomenclature. Be consistent. Don’t mix both styles.

 

- Results and Discussions:
  - “Proximate analyses, EC, pH and elemental analyses” section:
    - You should relate the higher ash content of the biochar to the initial ash content of the sludge.
    - As an explanation of the high ash content in faecal sludge, the disposal of liquid and solid waste in the toilets could also contribute to the high amount of inorganic material in the sludge.
    - It would have been interesting to measure the carbon content in the sludge to verify if this parameter increases or decreases after pyrolysis in your case.

 

-
  - “Fourier transform infrared (FTIR) spectroscopy” section:
    - You could conduct an element analysis of the ash from the three biochar samples and correlate the FTIR results to these results.
    - What could be the reasons that the sludge biochar have the same composition according to the FTIR spectra? What are the implications of this in the application?
    - There is not a clear difference between Figure 2 (biochar) and 3 (ashed-biochar). It seems to me that biochar and ashed-biochar are the same. Please clarify.
    - Paragraph 2: in Figure 2, 3 and 4, we cannot see the spectrum in the 3800 cm-1 to 3600 cm-1 region (related to OH group variations within mineral matter).
    - Paragraph 4: it is not clear if the discussion corresponds to the ashed or deashed biochar.
    - Paragraph 6: it is a repetition of Paragraph 5, so remove it.
    - Paragraph 8: the third sentence repeats information previously said.

 

-
  -  “Surface area” section:
    - Paragraph 2: it contradicts what has been stated in the previous paragraph. If the sorption isotherms are of Type II, it could be expected a nonporous and microporous adsorbent, but it was found that the biochar samples have a degree of microporosity and mesoporosity. Please clarify.
    - Paragraph 3: indicate to what corresponds “H3” and “H4”.
    - Paragraph 7: there is a lack of consistency in the notations of the biochar samples (e.g., instead of writing “WGL_BC” you wrote “WGL Biochar”). Correct this in the manuscript.
    - Paragraph 7: Can the difference of porosity between samples be related to their ash content?
    - Paragraph 8: The adsorption of water in biochar could follow different mechanism than N2. It would have been interesting to determine the sorption isotherm with water (in a Dynamic Vapor Sorption or using the saturated salt solution), in order to have a more realistic assessment of the water holding capacity in the biochar. If you add this data, your paper will take an extra dimension, making it more appealing.
    - In Figure 7, for better clarity, be consistent with the units between the graph and the legend (use nm or A, but avoid mixing them).
    - In Figure 7a, the WGL_BC peak is the highest in the micropore region, whereas it is the lowest in Figure 7b. Please clarify.

 

-
  - “X-ray diffraction (XRD) section”:
    - Paragraph 1: “Anhydrite” must be written without capital letter.
    - Paragraph 2: Where we can see the carbon fraction peaks in the XRD graphs? Please include an indication in Figure 9.
    - Paragraph 2, sentence 5: what do you refer to with “these peaks”?
    - Paragraph 3: How were the high content of the biochar samples in Ca, Si and K, as well as its high alkalizing capacity, determined?
    - Paragraph 3: “Thermonatrite” must be written without capital letter.

 

- Conclusion:
  - Indicate the weak points of using biochar derived from faecal sludge as soil condition, and suggest methods to overcome these weaknesses.
  - Precis in which context the high alkalinity of the biochar would be beneficial (i.e., acidic soils).

Is the work clearly and accurately presented and does it cite the current literature?

Yes

If applicable, is the statistical analysis and its interpretation appropriate?

Partly

Are all the source data underlying the results available to ensure full reproducibility?

Yes

Is the study design appropriate and is the work technically sound?

Yes

Are the conclusions drawn adequately supported by the results?

Yes

Are sufficient details of methods and analysis provided to allow replication by others?

Yes

Reviewer Expertise:

sludge and wastewater treatment, thermal process, biochar

I confirm that I have read this submission and believe that I have an appropriate level of expertise to confirm that it is of an acceptable scientific standard.

# Response

**Published:** 2022-11-30

**Authors:** Hannah Larissa Nicholas

**Affiliations:** Swansea University, UK

Keywords:

- **Comment 1**: Some keywords are redundant: “faecal sludge” and “fecal”. Remove one of the two.

**Authors response:** For some reason I cannot amend keywords on the system.

 Introduction:

- **Comment 2: **Paragraph 6: there are more advantages of the use of biochar as soil conditioner that you can mention (e.g., removal of pollutants, reduction of pollution of underground water, support the growth of microbial communities beneficial for plant growth, retention of nutrients).

**Authors response: **Agreed and added a section about this.

- **Comment  3: **Paragraph 6: concerning the literature about faecal sludge derived biochar, you should provide more details of what has been already studied, the objectives of the studies and the identified gaps (areas of knowledge not yet covered).

**Authors response: **Agreed. Another section with more details has been added to the introduction

**Material and Methods:**

 Biochar preparation:

- **Comment 4: **Indicate the conventional characteristics of the faecal sludge samples (i.e., moisture content, total solids, suspended solids, volatile solids, ash, COD, pH, electrical conductivity);

**Authors response: **Unfortunately, I do not have this data.

- **Comment 5: **With “solar drying”, do you mean “drying beds”? Be more specific.

**Authors response: **Agreed and added more specific terms.

- **Comment 6: **Provide more information about the pyrolyzers (estimated heating rate, throughput, residence time, carrier gas, etc...).

**Authors response: **I have added the data that I have available to me and added a photograph.

**Characterization of biochar:**

- **Comment 7: **For all tests, indicate the number of replicates and the statistical method to measure experimental uncertainty.

**Authors response:** I have added more detail in the method section and confirmed that standard deviation values have been provided for experimental uncertainty

- **Comment 8: **Mention explicitly that you characterized the biochar collected from the pyrolyzer.

**Authors response: **Agreed and amended.

- **Comment 9: **“Proximate analysis” section: for moisture content determination, be more precise on the duration of the samples in the oven.

**Authors response: **This has been amended in the text.

- **Comment 10: **“FT-IR analysis section”, paragraph 1, line 4: “infrared” must be written without capital letter.

**Authors response: **This has been amended in the text.

- **Comment 11: **“Surface area section”, paragraph 2: Was the degassing done at 130°C or 105°C? It's not clear.

**Authors response: **Changed and made clearer.

- **Comment 12: **“Cation exchange capacity” section: you wrote the name of the chemical elements in full letter and by the chemical nomenclature. Be consistent. Don’t mix both styles.

**Authors response: **This has been amended in the text.

**Results and Discussions:**

- “Proximate analyses, EC, pH and elemental analyses” section:

- **Comment 13: **You should relate the higher ash content of the biochar to the initial ash content of the sludge.-

**Authors response: **Agreed and amended.

- **Comment 14: **As an explanation of the high ash content in faecal sludge, the disposal of liquid and solid waste in the toilets could also contribute to the high amount of inorganic material in the sludge. It would have been interesting to measure the carbon content in the sludge to verify if this parameter increases or decreases after pyrolysis in your case.

**Authors response: **Agreed and amended.

 “Fourier transform infrared (FTIR) spectroscopy” section:

- **Comment 15: **You could conduct an element analysis of the ash from the three biochar samples and correlate the FTIR results to these results.

**Authors response:  **This would have been a really good idea. Unfortunately, as I have finished my PhD, I cannot do any further experimental work. Also, I didn’t have access to an elemental analyser so samples had to be sent away for analysis which makes it a bit trickier.

- **Comment 16: **What could be the reasons that the sludge biochar have the same composition according to the FTIR spectra? What are the implications of this in the application?

**Authors response: **I think the homogeneous nature of faecal sludge leads to the same composition and the similar temperatures that the sludge was pyrolzed at.

- **Comment 17: **There is not a clear difference between Figure 2 (biochar) and 3 (ashed-biochar). It seems to me that biochar and ashed-biochar are the same. Please clarify.-

**Authors response: **They are different, although not massively due to the very high ash content of the biochars. There is a low intensity peak in biochar between 1540 and 1650 whereas this peak is not evident in the ashed biochar (Figure 3.).

- **Comment 18: **Paragraph 2: in Figure 2, 3 and 4, we cannot see the spectrum in the 3800 cm-1 to 3600 cm-1 region (related to OH group variations within mineral matter). –

 
**Authors response: **I did cut that out to make the graph clearer but there were no peaks in that region. I would like to keep the graph as it is.

 - **Comment 19: **Paragraph 4: it is not clear if the discussion corresponds to the ashed or deashed biochar.

**Authors response: **I have amended this to make it clearer.

- **Comment 19: **Paragraph 6: it is a repetition of Paragraph 5, so remove it.

**Authors response**: Amended- thanks for spotting this.

- **Comment 20: **Paragraph 8: the third sentence repeats information previously said. –

 Not sure I can find this section. So, no action has been taken.

  “Surface area” section:

- **Comment 21: **Paragraph 2: it contradicts what has been stated in the previous paragraph. If the sorption isotherms are of Type II, it could be expected a nonporous and microporous adsorbent, but it was found that the biochar samples have a degree of microporosity and mesoporosity. Please clarify. –

**Authors response: **So later on we do clarify that the isotherms are pseudo type II. This is actually quite tricky.  The shape of the adsorption isotherms is best suited to type II, but type II isotherms are characterized by a lack of hysteresis and there is hysteresis to some degree here, especially when looking at NSP and WAI biochars. Type IIs are characteristic for nonporous and macroporous adsorbents. As there are indications of hysteresis loops especially for WAI and NSP biochar, this would indicate a type IV isotherm according to the IUPAC classification but there is no plateau characteristic for this type.

 So really, I can’t say its type II or type IV. According to (Sing and Williams, 2004) the isotherms can be classified as pseudo-type II, describing delayed capillary condensation due to the small rigidity of the aggregate structure of the adsorbent. So, I have essentially concluded it is a pseudo type II isotherm. I hope this makes sense!

- **Comment 22: **Paragraph 3: indicate to what corresponds “H3” and “H4”.

**Authors response: **I have made this much clearer.

- **Comment 23: **Paragraph 7: there is a lack of consistency in the notations of the biochar samples (e.g., instead of writing “WGL_BC” you wrote “WGL Biochar”). Correct this in the manuscript.

**Authors response: **This has been corrected.

- **Comment 24: **Paragraph 7: Can the difference of porosity between samples be related to their ash content?

**Authors response:  **Yes absolutely – I have added a paragraph at the end.

- **Comment 25: **Paragraph 8: The adsorption of water in biochar could follow different mechanism than N2. It would have been interesting to determine the sorption isotherm with water (in a Dynamic Vapor Sorption or using the saturated salt solution), in order to have a more realistic assessment of the water holding capacity in the biochar. If you add this data, your paper will take an extra dimension, making it more appealing.

**Authors response:  **This is really interesting! Unfortunately, I have completed my PhD and am unable to perform any additional experiments, however, I hope to keep working in this field so this is something I shall remember for the future.

- **Comment 26: **In Figure 7, for better clarity, be consistent with the units between the graph and the legend (use nm or A, but avoid mixing them).

**Authors response: **Agreed and amended.

- **Comment 27: **In Figure 7a, the WGL_BC peak is the highest in the micropore region, whereas it is the lowest in Figure 7b. Please clarify.

**Authors response: **Sorry, this is confusing, it is the highest peak at but a smaller value then Figure 7b. So, the y axis is smaller by a factor of 10 approximately for figure 7a then 7b. Then for WGL-BC it is highest in the mesopore region - figure 7a. It is lowest in the micropore region in figure 7b. I think as it has higher ash content the ash blocks access to the micropores. So, for WGL_BC the pore volume comes from the mesopore region. For NSP and WAI biochars their pore volume comes from the micropore region. So WGL_BC has a higher peak in the mesopore region (Fig 7a) – its pore volume comes from bigger pore sizes and a lower peak in the micropore region due to ash blocking the micropores, so it has limited pore volume arising from the micropore region.  Apologies if I have explained this terribly.

 “X-ray diffraction (XRD) section”:

- **Comment 28: **Paragraph 1: “Anhydrite” must be written without capital letter.

**Authors response: **Amended.

- **Comment 29: **Paragraph 2: Where we can see the carbon fraction peaks in the XRD graphs? Please include an indication in Figure 9.

**Authors response: ** This section has been taken out entirely - the links to the carbon fraction peak is tenuous, there may be a tail-end of a peak visible, further experiments with 2θ <20 would be needed to be able to positively identify any peaks and write confidently about them. The XRD results are still useful, and there is a lack of carbon fraction peaks in the spectra

- **Comment 30: **Paragraph 2, sentence 5:

**Authors response: ** clarified as per previous comment.

- **Comment 31: **Paragraph 3: How were the high content of the biochar samples in Ca, Si and K, as well as its high alkalizing capacity, determined?

**Authors response: ** Agreed that this wasn’t determined clearly enough, so this section has been removed

- **Comment 32: **Paragraph 3: “Thermonatrite” must be written without capital letter.

 
**Authors response: ** Amended.

 Conclusion:

- **Comment 33: **Indicate the weak points of using biochar derived from faecal sludge as soil condition and suggest methods to overcome these weaknesses.

**Authors response: ** Agreed this has been added to conclusion.

- **Comment 34: **Precis in which context the high alkalinity of the biochar would be beneficial (i.e., acidic soils).

**Authors response: ** Agree and have added a paragraph to the conclusion
